# Human sFLT1 Leads to Severe Changes in Placental Differentiation and Vascularization in a Transgenic hsFLT1/rtTA FGR Mouse Model

**DOI:** 10.3389/fendo.2019.00165

**Published:** 2019-03-21

**Authors:** Rebekka Vogtmann, Elisabeth Kühnel, Nikolai Dicke, Rikst Nynke Verkaik-Schakel, Torsten Plösch, Hubert Schorle, Violeta Stojanovska, Florian Herse, Angela Köninger, Rainer Kimmig, Elke Winterhager, Alexandra Gellhaus

**Affiliations:** ^1^Department of Gynecology and Obstetrics, University Hospital Essen, University Duisburg-Essen, Essen, Germany; ^2^Department of Developmental Pathology, Institute of Pathology, University Medical School, Bonn, Germany; ^3^Department of Obstetrics and Gynecology, University Medical Center Groningen, University of Groningen, Groningen, Netherlands; ^4^Experimental and Clinical Research Center, Charité Medical Faculty, and the Max Delbrück Center for Molecular Medicine in the Helmholtz Association, Berlin, Germany; ^5^EM Unit, Imaging Center Essen, University Hospital Essen, University Duisburg-Essen, Essen, Germany

**Keywords:** human sFLT1, fetal growth restriction, vascularization, placenta, transgenic mouse model

## Abstract

The anti-angiogenic soluble fms-like tyrosine kinase 1 (sFLT1) is one of the candidates in the progression of preeclampsia, often associated with fetal growth restriction (FGR). Therapeutic agents against preeclampsia with/without FGR, as well as adequate transgenic sFLT1 mouse models for testing such agents, are still missing. Much is known about sFLT1–mediated endothelial dysfunction in several tissues; however, the influence of sFLT1 on placental and fetal development is currently unknown. We hypothesize that sFLT1 is involved in the progression of FGR by influencing placental differentiation and vascularization and is a prime candidate for interventional strategies. Therefore, we generated transgenic inducible human sFLT1/reverse tetracycline-controlled transactivator (hsFLT1/rtTA) mice, in which hsFLT1 is ubiquitously overexpressed during pregnancy in dams and according to the genetics in hsFLT1/rtTA homozygous and heterozygous fetuses. Induction of hsFLT1 led to elevated hsFLT1 levels in the serum of dams and on mRNA level in all placentas and hetero-/homozygous fetuses, resulting in FGR in all fetuses at term. The strongest effects in respect to FGR were observed in the hsFLT1/rtTA homozygous fetuses, which exhibited the highest hsFLT1 levels. Only fetal hsFLT1 expression led to impaired placental morphology characterized by reduced placental efficiency, enlarged maternal sinusoids, reduced fetal capillaries, and impaired labyrinthine differentiation, associated with increased apoptosis. Besides impaired placental vascularization, the expression of several transporter systems, such as glucose transporter 1 and 3 (*Glut-1*; *Glut-3*); amino acid transporters, solute carrier family 38, member one and two (*Slc38a1*; *Slc38a2*); and most severely the fatty acid translocase *Cd36* and fatty acid binding protein 3 (*Fabp3*) was reduced upon hsFLT1 expression, associated with an accumulation of phospholipids in the maternal serum. Moreover, the Vegf pathway showed alterations, resulting in reduced Vegf, Vegfb, and Plgf protein levels and increased Bad and Caspase 9 mRNA levels. We suggest that hsFLT1 exerts an inhibitory influence on placental vascularization by reducing Vegf signaling, which leads to apoptosis in fetal vessels, impairing placental differentiation, and the nutrient exchange function of the labyrinth. These effects were more pronounced when both the dam and the fetus expressed hsFLT1 and ultimately result in FGR and resemble the preeclamptic phenotype in humans.

## Introduction

Appropriate development and growth of the fetus depend on adequate vascularization of both fetus and mother at the feto-maternal unit; this vascularization involves uterine vasodilation and vessel remodeling upon trophoblast invasion, as well as vasculo- and angiogenesis within the placenta. The consequences of abnormal vascular development have been associated with various pregnancy-related pathologies, ranging from miscarriage to fetal growth restriction (FGR) or preeclampsia (PE) (1).

At least 60% of the 4 million neonatal deaths that occur worldwide each year are associated with low birth weight caused by FGR, which is characterized by insufficient growth during pregnancy or preterm delivery (2). Epidemiologic studies have found that children born with FGR are a risk cohort with increased incidence and prevalence of diseases such as short- and long-term metabolic and cardiovascular alterations, adiposity, and neurological disorders in later life (3–5). Currently there are no treatments for FGR.

FGR is one of the main consequences of the pregnancy disorder PE, which is characterized by maternal hypertension and proteinuria. PE is still a leading cause of maternal and neonatal mortality (6, 7) and is often associated, amongst other factors such as oxidative stress and genetic factors, with an overexpression of the angiogenesis inhibitor soluble fms-like tyrosine kinase 1 (sFLT1) in the placenta as the pregnancy progresses (7, 8). sFLT1 is a soluble splice variant of the membranous vascular endothelial growth factor receptor 1 (VEGFR-1), which contains only the extracellular domain. Therefore, sFLT1 acts as a decoy receptor by binding and reducing free circulating levels of the angiogenesis-promoting VEGF and placental growth factor (PlGF), thereby limiting their bioavailability. There is strong evidence that overproduction of sFLT1 in the placenta and the resulting high levels of sFLT1 in maternal serum are an important cause of vascular dysfunction associated with PE through sFLT1–dependent antagonism of VEGF (8, 9). The linkage of sFLT1 with the pathophysiology of PE was clearly shown by a study of Levine et al. (10), which found that an increase in circulating levels of sFLT1 is associated with the severity of PE. Currently the sFLT1/PlGF ratio is used as a clinical biomarker for predicting PE (11–13). In addition, an elevated level of sFLT1 leads to several maternal consequences, such as endothelial dysfunction that causes hypertension and “glomerular endotheliosis” that finally leads to cellular injury and disruption of the filtration apparatus, with proteinuria, and edema as consequences (8).

We recently confirmed the importance of sFLT1 in human pregnancy and as a clinical marker for early and late-onset PE, as well as FGR (14). This role has been further confirmed by the studies of Thadhani et al. (15, 16), showing that therapeutic apheresis, which reduces the circulating levels of sFLT1 and the severity of proteinuria in women with exceedingly preterm PE, appears to prolong pregnancy without severe adverse consequences to the mother or the fetus. Much is known about the molecular mechanisms of sFLT1, which disturbs endothelial cell function [reviewed by Lecarpentier and Tsatsaris (17)]; however, the way in which sFLT1 affects the placenta and fetus, resulting in FGR, is currently unknown. In own previous studies, using a lentiviral placenta-specific sFLT1 mouse model for FGR and PE [established by Kumasawa et al. (18)], we observed a reduction in fetal and placental weight associated with a reduction in the transporting trophoblast layer and by changes in the expression of labyrinthine nutrient transporters [(19); reviewed in Winterhager and Gellhaus (20)]. These findings demonstrate that sFLT1 not only acts directly on endothelial cells and changes endothelial physiology but also directly or indirectly affects placental development and function.

Studies using several animal models that overexpress human sFLT1 (hsFLT1) have shown that hsFLT1 causes symptoms of PE, such as hypertension and proteinuria (8). Since most of the existing PE mouse models were developed by injection of replication-deficient sFLT1 lenti- or adenoviruses, we developed doxycycline (Dox)-inducible transgenic hsFLT1 mice [hsFLT1/reverse tetracycline-controlled transactivator (rtTA) mice] to receive a stable and reproducible hsFLT1 expression. In this study we found ubiquitous maternal and placental/fetal expression of hsFLT1 or ubiquitous maternal expression only upon treatment with Dox (Tet-On System induced during midgestation). Thus, we can discriminate between the consequences of maternally expressed hsFLT1 and those of maternally and placentally/fetally expressed hsFLT1 on placental and fetal outcome. We hypothesize that elevated levels of hsFLT1, if increased in all three compartments (dam, placenta, and fetus) may influence placental function and transport capability via altered placental angiogenesis and altered Vegf signaling caused by reduced levels of growth factors such as Vegf and Plgf, thereby resulting in FGR.

## MaterialS and Methods

### Generation of hsFLT1/rtTA Mice and Experimental Procedures

**Figure 1:**
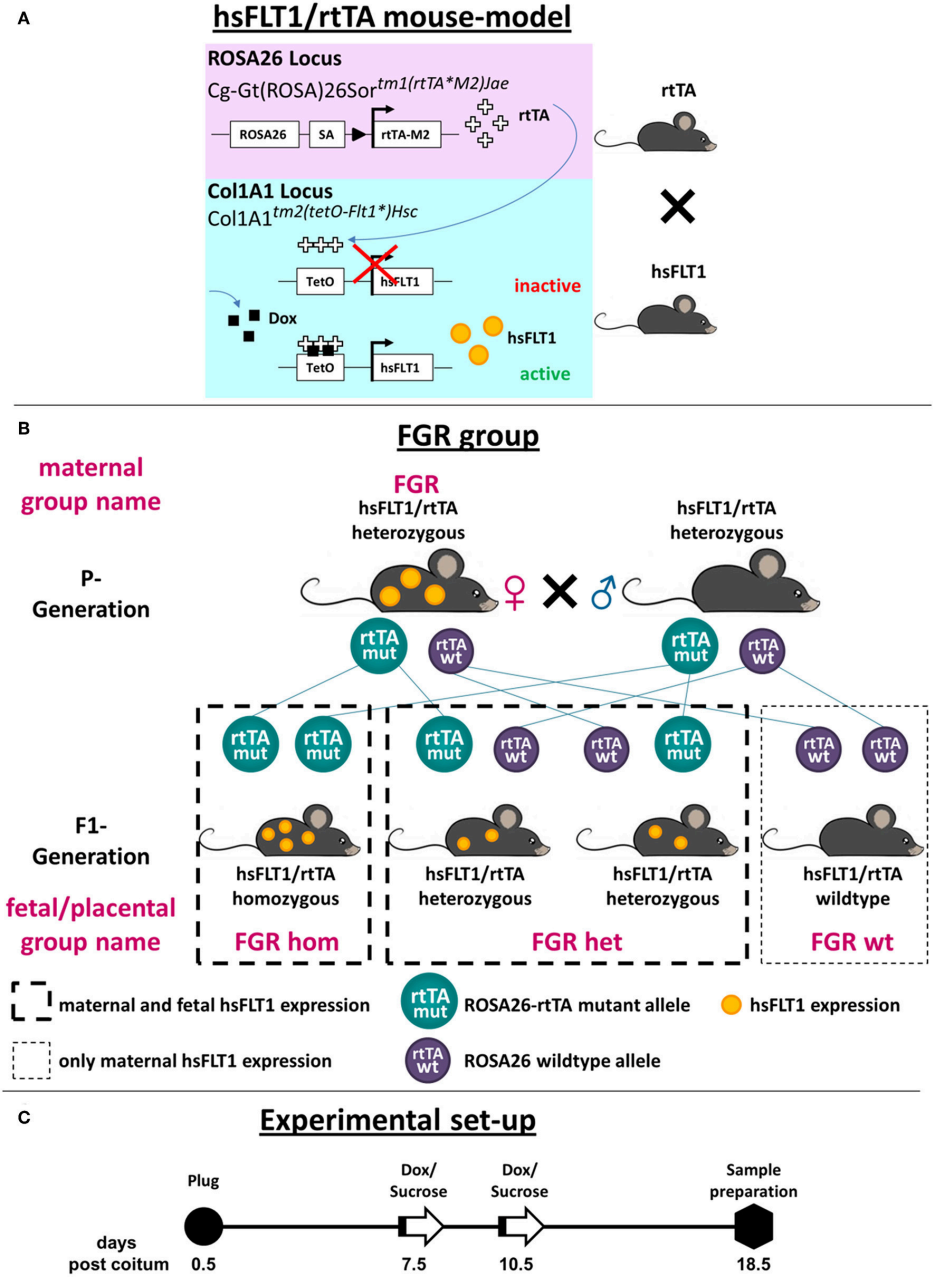
Generation of the hsFLT1/rtTA mouse model with ubiquitous overexpression of hsFLT1. **(A)** We mated single transgenic Gt(ROSA)26Sor^*tm*1(rtTA^*^M2)*Jae*^ mice with single transgenic Col1a1^*tm*2(*tetO*−*Flt*1^*^)*Hsc*^ mice to generate double transgenic human soluble fms-like tyrosine kinase 1 reverse tetracycline-controlled transactivator (hsFLT1/rtTA) mice. These mice ubiquitously express rtTA, which induces hsFLT1 expression upon treatment with doxycycline (Dox): hsFLT1/rtTA+Dox/FGR (fetal growth restriction) group. Without Dox, no expression of hsFLT1 occurred (hsFLT1/rtTA-Dox/control). Single transgenic hsFLT1 mice treated with Dox (hsFLT1+Dox/Dox control) cannot express hsFLT1 but were used as a control for Dox effects (scheme adapted from that of Hubert Schorle, Bonn). **(B)** Mating scheme of FGR group. Heterozygous sFlt-1/rtTA mice were mated. Since Dox passes the placental barrier, hsFLT1/rtTA homozygous (hom), and heterozygous (het) fetuses expressed hsFLT1, whereas wild-type (wt) fetuses did not. For this reason the FGR experimental group was subdivided into FGR hom, FGR het, and FGR wt (depending on the *rtTA* genotype) for fetal and placental analysis. P, parental; F1, first filial generation of fetuses. **(C)** Experimental set-up. Mice were mated overnight. The day after the development of a vaginal plug was defined as day 0.5 post coitum (dpc). At early to midgestation (7.5 or 10.5 dpc), dams were treated either with 2 mg Dox and 3% [w/v] sucrose per ml of drinking water or with 3% [w/v] sucrose only as a control until cesarean section and sample preparation were performed at 18.5 dpc (cesarean section and sample preparation).

A mouse strain harboring a tetracycline-inducible cassette expressing hsFLT1 was generated according to previously published protocols (21, 22). For details of the generation of the hsFLT1/rtTA mouse and the experimental set-up, see Figure 1A. The mice were generated on a 129/Sv background. The full-length *hsFLT1* cDNA (2100 bp) was inserted into pBS31_tetO_promoter/simian virus 40 5′ of the tetO minimal promoter of cytomegalovirus (CMV). KH2 embryonic stem cells (ESCs) carrying the *rtTA* transgene in the ROSA26 locus were electroporated with 50 μg of pBS31_*hsFLT1* and 25 μg of an expression vector for Flp recombinase [pCAGGS-*flpE;* (23)]. The KH2 ESCs originated from the v6.5 mouse ESC line, which was established from cells derived from the inner cell mass (ICM) of a 3.5-day-old mouse embryo from a C57BL/6 × 129/sv cross. Flp-mediated recombination of pBS31_*hsFLT1* leads to the integration of *hsFLT1* cDNA into the ColA1 locus of KH2 ESCs. This recombination initiates the expression of the promoter- and ATG-less hygromycin resistance cassette present in the Col1A1 locus. One day after electroporation, 140 μg/ml hygromycin was added; colonies were selected after 10 days; and clones were screened by Southern blotting using SpeI to digest genomic DNA and a 3′ internal probe (21). Five positive clones in which hsFLT1 expression was induced after the addition of 0.5 μg/ml Dox for 48 h were expanded. The resulting *hsFLT1–*KH2 ESCs were treated with Dox (1 mg/ml), and the cell culture supernatants were analyzed for hsFLT1 secretion with enzyme-linked immunosorbent assay [ELISA; human sVEGFR-1 Quantikine ELISA (SVR100B), R&D Systems, Minneapolis, MN, USA].

Subsequently, *hsFLT1*–KH2 ESCs were injected into blastocysts isolated from 129/Sv Jae female mice; the blastocysts were then transplanted into pseudopregnant mice. We obtained 20 chimeric mice and achieved germline transmission. The newly generated mice were registered with the mouse genome informatics database; the allele was named Col1A1^*tm*2(*tetO*−*Flt*1^*^)*Hsc*^ (MGI: 6202353). These mice were mated with Rosa26–*rtTA-M2* mice (B6.Gt(ROSA)26Sor^*tm*1(rtTA^*^M2)*Jae*^, Stock No. 006965; Jackson Laboratory, Bar Habor, ME, USA) to generate hsFLT1/rtTA double transgenic mice. The Rosa26 gene locus is ubiquitously expressed; the reverse tetracycline-controlled transactivator protein (rtTA-M2) therefore reveals nearly ubiquitous target gene expression in many tissues. According to the Jackson Laboratory, expression can be observed in liver, bone marrow, stomach, intestine, and skin, with lower levels in the heart, lungs, kidney, spleen, and thymus and no expression in the brain and testes (https://www.jax.org/strain/006965).

In the parental generation (P generation) of double transgenic hsFLT1/rtTA mice, ubiquitously expressed rtTA induces hsFLT1 expression upon treatment with Dox. When Dox is added, rtTA can bind to the TetO promoter of the *hsFLT1* transgene, leading to hsFLT1 expression (hsFLT1/rtTA+Dox/FGR group); without Dox, hsFLT1 is not expressed (hsFLT1/rtTA-Dox/control group). Single transgenic hsFLT1 mice treated with Dox do not express hsFLT1 but can be used as a control for the effects of Dox (hsFLT1+Dox/Dox control group). Since Dox passes the placental barrier, hsFLT1/rtTA homozygous (hom) and heterozygous (het) fetuses/placentas in the first filial generation (F1 generation) of the FGR group can also express hsFLT1, whereas wild-type (wt) fetuses/placentas cannot, because they do not express rtTA protein and thus the Tet-On system does not function (Figure 1A). Therefore, the maternal FGR group must be subdivided by the fetal *rtTA* genotype for placental and fetal analysis (Figure 1B). This process leads to the experimental groups shown in Table 1.

**Table 1:** Maternal and fetal experimental groups.

**Maternal groups**	**Fetal groups**
FGR (hsFLT1 expression)	FGR hom (maternal and fetal hsFLT1 expression)
	FGR het (maternal and fetal hsFLT1 expression)
	FGR wt (only maternal hsFLT1 expression)
Control (no hsFLT1 expression)	Control (no hsFLT1 expression)
Dox control (no hsFLT1 expression)	Dox control (no hsFLT1 expression)

*Dox, doxycycline; FGR, fetal growth restriction; hsFLT1, human soluble fms-like tyrosine kinase 1*.

For experimental procedures, animals were mated overnight. When a vaginal plug was present, the following day was counted as gestational day 0.5 (day post coitum; dpc). Beginning at early to midgestation (7.5 or 10.5 dpc) until the end of pregnancy (18.5 dpc), animals were treated with either Dox and sucrose or sucrose only (Figure 1C). Therefore, the FGR experimental group and the Dox control group received 2 mg/ml Dox [0.2% (w/v); Merck, Darmstadt, Germany] and 30 mg/ml sucrose [3% (w/v); Carl Roth, Karlsruhe, Germany] in the drinking water, whereas the control group received only 30 mg/ml sucrose in the drinking water. The drinking water was renewed every third day. The endpoint of the experiments was set at 18.5 dpc. Mice from each experimental group were sacrificed; whole blood was collected by cardiopuncture; and fetuses, placentas, and maternal organs such as liver and kidneys were isolated and weighed. Fetal maturation, including FGR characteristics, was assessed on the basis of morphological criteria according to the Theiler developmental atlas: (http://www.emouseatlas.org/emap/ema/theiler_stages/house_mouse/book.html). The fetal to placental weight ratio was determined because it reflects the efficiency of the placenta in meeting the nutritional demands of the growing fetus (24).

All mice were housed in the animal facility of the University Hospital Essen in a specific pathogen–free environment and were exposed to cycles of 12 h of light and 12 h of dark. They were provided with food and water *ad libitum*. All animal procedures were performed in accordance with the German laws for animal protection (No.: G1265/12 and G1644/17).

### Tissue Preparation

At 18.5 dpc, anesthetized pregnant mice were killed by cervical dislocation. Maternal blood was collected; maternal liver, and kidney, as well as fetuses and placentas, were dissected in sterile phosphate-buffered saline (PBS); and the amniotic membrane was removed from the placenta. Isolated fetuses and placentas were weighed with an ALJ 220-4 NM analytical balance (Kern, Ebingen, Germany) with a linearity of ±0.2 mg. Organs were either frozen and stored at −80°C (for RNA, DNA, and protein analysis) or fixed in 4% paraformaldehyde (PFA) for 24 h and stored in 70% ethanol until being embedded in paraffin standard procedures (for morphology).

### Genomic DNA Isolation, Genotyping, and Sex Determination

Genomic DNA was isolated from ear punch tissue samples with the REDExtract-N-Amp™ Tissue PCR Kit (Sigma-Aldrich, St. Louis, MO, USA) according to the manufacturer's protocol. The quality and quantity of DNA were verified with μCuvette G1.0 and BioPhotometer Plus (Eppendorf, Hamburg, Germany). Genotyping and sex determination of mice were performed with a standard PCR program (hsFLT1: initial denaturation 95°C, 5 min; 40 cycles 94°C, 45 s, 60°C, 45 s, 72°C, 1 min, final extension 72°C, 5 min; rtTA: initial denaturation 94°C, 3 min; 35 cycles 94°C, 45 s, 65°C, 1 min., 72°C, 1 min, final extension 72°C, 2 min) and the appropriate primers (Table 2).

**Table 2:** Oligonucleotides used for genotyping, sex determination and gene expression analysis in hsFLT1/rtTA mouse model.

**Gene**	**NCBI number**	**Primer sequence (5′ → 3′)**	**Product length (bp)**
**GENOTYPING**			
*hsFLT1*	XM_017020485.1	for: AATCATTCCGAAGCAAGGTG	221
		rev: TTTCTTCCCACAGTCCCAAC	
*rtTA*		for: AAAGTCGCTCTGAGTTGTTAT	
		rev-wt: GGAGCGGGAGAAATGGATATG	650
		rev-mut: GCGAAGAGTTTGTCCTCAACC	340
**SEX DETERMINATION**			
*IL-3*	NM_010556.4	for: GGGACTCCAAGCTTCAATCA	544
		rev: TGGAGGAGGAAGAAAAGCAA	
*Sry*	NM_011564.1	for: TGGGACTGGTGACAATTGTC	402
		rev: GAGTACAGGTGTGCAGCTCT	
**REFERENCE/HOUSEKEEPING GENE**			
*Gapdh*	XM_011241214.1	for: ACAACTCACTCAAGATTGTCAGCA	121
		rev: ATGGCATGGACTGTGGTCAT	
**AMINO ACID TRANSPORTERS**			
*Slc38a1*	NM_001166458.1	for: AGCACAGGCGACATTCTCATC	133
		rev: ACAGGTGGAACTTTGTCTTCTTG	
*Slc38a2*	NM_175121.3	for: ACAAATGGGTTGTGGTATCTG	92
		rev: CCTAGATTTCTCAGCAGTGACAATG	
**FATTY ACID TRANSPORTERS**			
*Cd36*	XM_006535623.2	for: CAGTGCAGAAACAATGGTTGTCT	137
		rev: TGACATTTGCAGGTCTATCTACG	
*Fabp3*	NM_010174.1	for: CTGTCACCTCGTCGAACTCT	166
		rev: TTTGTCGGTACCTGGAAGCT	
**GLUCOSE TRANSPORTERS**			
*Glut-1*	NM_011400.3	for: GCTGTGCTTATGGGCTTCTC	202
		rev: ACACCTGGGCAATAAGGATG	
*Glut-3*	NM_011401.4	for: GGAGGAGAACCCTGCATATGATA	96
		rev: TGGCTTCATAGTCATCCTTTAGTAAC	
**LABYRINTHINE DIFFERENTIATION MARKERS**			
*Cx26*	NM_008125.3	for: ATGCTACGACCACCACTTCC	194
		rev: TACGGACCTTCTGGGTTTTG	
*Gcm1*	NM_008103.3	for: TGCTCACCTATGGCTCTCCT	201
		rev: AAAATTCTGCCSAGCCCTTT	
**FETAL ENDOTHELIAL CELL MARKER**			
*Cd31*	NM_008816.3	for: ATGACCCAGCAACATTCACA	200
		rev: CACAGAGCACCGAAGTACCA	
**TROPHOBLAST GIANT CELL MARKERS**			
*Ctsq*	NM_029636	for: GTGATCTGAGGCAGTAGTGGTC	180
		rev: GTACTTCTTCCTCCGGACTGTATA	
*PLAP*	XM_006538500.2	for: TGAGGGCAATGAGGTCACAT	161
		rev: CCTCTGGTGGCATCTCCTTA	
*Prl3d1*	NM_008864	for: TGGAGCCTACATTGTGGTGGA	131
		rev: TGGCAGTTGGTTTGGAGGA	
*Prl3b1*	NM_008865.3	for: AGCAGCCTTCTGGTGTTGTC	197
		rev: TGTGACACCACAATCACACG	
*Prl2c2*	NM_011118	for: AGGAGCCATGATTTTGGATG	203
		rev: ACCAGGCAGGGTTCTTCTTT	
**SPONGIOTROPHOBLAST AND GLYCOGEN CELL MARKERS**			
*Cx31*	NM_008126	for: GTCTACTAGCGCTGGGATGG	227
		rev: GTGCCAAACCTTCTCATGGT	
*Cx31.1*	NM_008126	for: CCCTCTTTGCTTGTGGTCAT	151
		rev: CCTTGAACGAGAGGCTGAAG	
*Igf2*	NM_010514	for: CGTTTGGCCTCTCTGAACTC	155
		rev: GACGACTTCCCCAGATACCC	
*Pcdh12*	NM_017378.2	for: CTTCACCTCATCACGCTCAA	197
		rev: TGCCCTCTGTCCTCTGCTAT	
*Tpbpa*	NM_009411	for: CCAGCACAGCTTTGGACATCA	116
		rev: AGCATCCAACTGCGCTTCA	
**ANGIOGENESIS MARKERS**			
*Flk-1*	NM_001363216.1	for: GGCGGTGGTGACAGTATCTT	162
		rev: GTCACTGACAGAGGCGATGA	
*m(s)Flt-1*	NM_001363135.1	for: TATAAGGCAGCGGATTGACC	159
		rev: TCATACACATGCACGGAGGT	
*Flt-4*	NM_008029.3	for: GTGGCTGTGAAGATGCTGAA	199
		rev: TGACACGCAAGAAGTTGGAG	
*Plgf*	XM_011244016.1	for: CGTCCTGTGTCCTTCTGAGT	200
		rev: CCTCTTCCTCTTCCCCTTGG	
*Vegfa*	NM_001025257.3	for: CAGGCTGCTGTAACGATGAA	140
		rev: GCATTCACATCTGCTGTGCT	
*Vegfb*	NM_011697.3	for: AACACAGCCAATGTGAATGC	157
		rev: GGAGTGGGATGGATGATGTC	
*Vegfc*	NM_009506.2	for: CAAGGCTTTTGAAGGCAAAG	159
		rev: TCCCCTGTCCTGGTATTGAG	
*Vegfd*	NM_001308489.1	for: CAACAGATCCGAGCAGCTTC	155
		rev: AAAGTTGCCGCAAATCTGGT	
**PROAPOPTOTIC MARKERS**			
*Bad*	NM_001285453.1	for: GGAGCTTAGCCCTTTTCGAG	166
		rev: GCTTTGTCGCATCTGTGTTG	
*Casp9*	NM_001355176.1	for: GATGCTGTCCCCTATCAGGA	151
		rev: CGATGTACCAGGAGCCACTT	
**HYPOXIA-INDUCIBLE MARKERS**			
*Nos3*	XM_006535639.3	for: GACCCTCACCGCTACAACAT	209
		rev: CTGGCCTTCTGCTCATTTTC	
*Hif1α*	NM_001313920.1	for: TCAAGTCAGCAACGTGGAAG	198
		rev: TATCGAGGCTGTGTCGACTG	
*Hmox1*	NM_010442.2	for: CACGCATATACCCGCTACCT	175
		rev: CCAGAGTGTTCATTCGAGCA	
*Cited2*	NM_010828	for: CTAGGGCAGCGGAGGAAAAG	176
		rev: TTCTGCTCGGAACACCGAAG	

*A, adenine; Bad, Bcl-2–associated death promoter; bp, base pair; Casp9, caspase 9; Cd31, cluster of differentiation 31; Cited2, Cbp/P300 Interacting Transactivator With Glu/Asp Rich Carboxy-Terminal Domain 2; Ctsq, cathepsin Q; C, cytosine; Cx26, connexin 26; Fabp3, fatty acid binding protein 3; Flk-1, fetal liver kinase 1; Flt-4, Fms-like tyrosine kinase 4; for, forward; Gapdh, glyceraldehyde-3-phosphate dehydrogenase; Gcm1, glial cell missing 1; Glut-1, glucose transporter 1; G, guanine; Hif1α, hypoxia-inducible factor-1alpha; Hmox1, Heme Oxygenase 1; hsFLT1, human soluble fms-like tyrosine kinase 1; Igf2, insulin-like growth factor 2; IL-3, interleukin-3; mut, mutant; NCBI, National Center for Biotechnology Information; Nos3, Nitric Oxide Synthase 3; Pcdh12, Protocadherin 12; PLAP, Placental Alkaline Phosphatase; Plgf, Placental Growth Factor; Prl3b1, prolactin family 3; subfamily b, member 1; rev, reverse; rtTA, reverse tetracycline-controlled transactivator; Slc38a1, Solute Carrier Family 38 Member 1; Sry, sex determining region Y; Tpbpa, Trophoblast-specific protein alpha; T, thymine; Vegfa, Vascular Endothelial Growth Factor A; wt, wild-type*.

### Genomic DNA Isolation and Pyrosequencing

Approximately 20 mg of placental tissue was homogenized with a Tissue Lyser LT (Qiagen, Hilden, Germany). Genomic DNA was isolated with the AllPrep DNA/RNA Mini Kit (Qiagen) according to the manufacturer's protocol. The quality and quantity of DNA were verified with Nanodrop 2000c (Thermo Fisher Scientific, Pittsburgh, PA, USA). Bisulfite conversion of 500 ng genomic DNA was performed with the EZ DNA methylation gold kit (Zymo Research, Leiden, The Netherlands) according to the manufacturer's protocol. Pyrosequencing was performed as previously described by Freitag et al. (25). The sequences of bisulfite-specific primers for long interspersed element 1 (*LINE1*), insulin-like growth factor two (Igf2) differentially methylated region two (*Igf2-DMR2*), and H19 imprinting control region (*H19-ICR*) have been previously published (25). The PCR product was analyzed for the extent of methylation per selected CpG position with a Pyromark Q48 sequencer (Qiagen). Data were analyzed with PyroMark Q48 autoprep software (Qiagen). The level of DNA methylation was given as a percentage. Samples were obtained from complete placentas. The experimental groups FGR hom (*n* = 4), FGR het (*n* = 7), and FGR wt (*n* = 7) were analyzed, as well as the control group (*n* = 10) and the Dox control group (*n* = 6).

### RNA Extraction, cDNA Synthesis, and Quantitative PCR

Total RNA was extracted from ~10 mg frozen samples of placenta, liver, kidney, and fetus with the E.Z.N.A Total RNA Kit (Omega Bio-tek, Norcross, GA, USA) according to the manufacturer's protocol. Complementary DNA (cDNA) was synthesized with 1 μg RNA as previously described (19). Gene expression was measured from 1 μl cDNA with 19 μl of the VeriQuest® Fast SYBR® Green qPCR Mix (Affymetrix, Santa Clara, CA, USA) and the ABI Prism 7300 Sequence Detection System (Applied Biosystems, Foster City, CA, USA) with a standard PCR program (Table 2). For quantitative measurement, standard curves of 1 μl cDNA of standards with known concentrations from 1,000 to 0.1 fg of each measured gene were used. The quantitative PCR (qPCR) analyses were carried out in triplicate. The amount of cDNA in each sample was normalized to glyceraldehyde-3-phosphate dehydrogenase (*Gapdh*) as a housekeeping gene and experimental groups were normalized to control group. Primer sequences are listed in Table 2. We tested the following experimental groups: FGR hom *n* = 7, FGR het *n* = 15, control *n* = 12, Dox control *n* = 13.

### Analysis of hsFLT1 Serum Levels

Blood was collected from anesthetized pregnant mice by cardiopuncture after cervical dislocation. Serum samples were prepared by centrifuging clotted blood for 15 min at 3,000 g and 4°C; the serum was stored at −80°C until analysis. The undiluted sample was used to measure the concentration of hsFLT1 (BRAHMS sFlt-1 KRYPTOR assay) with a BRAHMS KRYPTOR compact PLUS analyzer based on time-resolved amplified cryptate emission (TRACE® technology; Thermo Fischer Scientific), according to the manufacturer's protocol (FGR *n* = 10, control *n* = 3, Dox control *n* = 6). The detection limit for hsFLT1 was assessed at 22 pg/ml. The sensitivity of the functional assay, detected by interassay precision of a 20% coefficient of variability (CV), has been assessed to be lower than 29 pg/ml for hsFLT1.

### Mouse Angiogenesis Antibody Array

Approximately 20 mg of frozen placenta was homogenized in radioimmunoprecipitation assay (RIPA) protein extraction buffer [50 mM Tris/HCl, 150 mM NaCl, 1% NP-40, 0.25% Na-deoxycholate, 1 mM ethylenediaminetetraacetic acid (EDTA)]. The protein content was determined with the Pierce BCA Protein Assay Kit (Thermo Scientific, Rockford, IL, USA). Murine angiogenesis-related proteins were simultaneously detected with a Proteome Profiler Angiogenesis Antibody Array according to the manufacturer's protocol (R&D Systems, Minneapolis, MN USA). In principle, selected capture antibodies for each of 53 different angiogenesis proteins have been spotted in duplicate on nitrocellulose membranes. For protein detection, a total of 50 μg protein of a pooled sample of each condition (control *n* = 5, Dox control *n* = 5, FGR het *n* = 5 and FGR hom *n* = 5; each 10 μg) was diluted and mixed with a cocktail of biotinylated detection antibodies. The sample/antibody mixture was then incubated with the array at 4°C over night. Streptavidin-horseradish peroxidase and chemiluminescent detection reagents were added, and chemiluminescence was detected with ChemiDoc™ XRS+ System (Bio-Rad Laboratories, Inc., Hercules, CA, USA). Pixel intensity for each spot was measured with ImageJ (National Institutes of Health, Bethesda, MD, USA) and normalized to negative and reference spots. Normalized intensities of the pair of duplicate spots representing each angiogenesis-related protein were determined and the most relevant proteins were presented.

### Serum Metabolome Detection

Maternal serum was obtained as described above. Serum metabolome analysis was performed by Biocrates with the Biocrates AbsoluteIDQ p180 Kit at their facility (Biocrates Life Sciences AG, Innsbruck, Austria), as described previously (26). The following maternal groups were analyzed: FGR group, *n* = 6 [hsFLT1/rtTA, treatment with Dox at 7.5 (*n* = 3) and 10.5 dpc (*n* = 3), respectively]; control group, *n* = 6 [hsFLT1/rtTA, not treated with Dox (*n* = 3) and hsFLT1, treated with Dox at 10.5 dpc (*n* = 3)]. A commercially available direct-flow injection system and a liquid chromatography tandem mass spectrometry (LC-MS/MS) kit was used to analyze 188 available metabolites in plasma samples, including hexose (1), amino acids (21), biogenic amines (21), glycerophospholipids (90), sphingolipids (15), and acylcarnitines (40). Internal standards were pipetted in advance, and a calibration standard mix in seven different concentrations was included in a standardized assay in a 96-well plate, with 10 μl serum in each well. Derivatization was performed with a 5% solution of phenyl isothiocyanate, followed by extraction via the addition of methanol with 5 mM ammonium acetate. The samples were analyzed with an API4000 Qtrap® MS/MS instrument (Applied Biosystems) using a reverse-phase high-performance liquid chromatography (HPLC) column, followed by a direct-flow injection assay.

### Histologic and Morphometric Analysis

Formalin-fixed and paraffin-embedded placentas were sectioned at 7 μm and mounted on either standard slides (Engelbrecht Medizin- und Labortechnik GmbH, Edermünde, Germany) or Superfrost Plus Slides (R. Langenbrinck, Emmendingen, Germany). For morphological analysis, sections were stained with hematoxylin and eosin (H&E). Stained slides were scanned with the Aperio CS2 ScanScope slide scanner (Leica, Wetzlar, Germany) at 40×, and images were converted to TIFFs via Image Scope (Version 12.3.2.8013; Leica). Scanned slides were opened by ImageJ with the plugin “bioformats_package.jar.”

Morphometric analysis of placental compartments (labyrinth and spongiotrophoblast layer) was performed on three serial sections of three different parts in the proximity of the umbilical cord from each experimental group (FGR wt *n* = 8, control *n* = 8, Dox control *n* = 7). Total placental area was calculated by combining measurements of labyrinth and spongiotrophoblast area; differences in placental compartment composition were measured by the ratio of labyrinth to spongiotrophoblast area as previously described (19).

### Immunohistochemical Analysis

Deparaffinized sections were used for immunostaining. Endogenous peroxidase was blocked with H_2_O_2_ in methanol (1 ml methanol per 25 μl H_2_O_2_). Antigens were retrieved by boiling sections with citrate buffer for 10 min. After blocking with bovine serum albumin, sections were incubated overnight at 4°C with rat anti-Cd31 (DIA310; 1:20; Dianova, Hamburg, Germany). Bound primary antibody was visualized with goat anti-rat immunoglobulin G horseradish peroxidase (IgG-HRP) secondary antibody (sc-2032; 1:100; Santa Cruz Biotechnology Inc., Santa Cruz, CA, USA) and the Liquid DAB+ Substrate Chromogen System (Dako, Carpinteria, CA, USA).

For placental alkaline phosphatase (PLAP) staining, samples were deparaffinized, rehydrated, and incubated with Nitro Blue Tetrazolium (NBT)/5-bromo-4-chloro-3-indolyl phosphate (BCIP) (Promega Corporation, Madison, WI, USA) as a substrate for PLAP. Nuclear Fast Red (Sigma-Aldrich) was used for counterstaining. Stained slides were scanned and converted as described above.

### Statistical Analysis

Normal distribution was tested with D'Agostino-Pearson omnibus K2 test and Shapiro-Wilk test. Both normality tests could not prove that all of our data were sampled from a Gaussian distribution. Therefore differences between groups were calculated with the Kruskal–Wallis test and Dunn's multiple comparison test. Data are either presented in mean ± standard error of mean or in box and whisker plot. For all statistical tests, a probability value (*p*-value) of 0.05 or less was indicated with ^*^, ^**^p < 0.01 ^***^p < 0.001. Outliers were detected performing Grubbs' test (https://www.graphpad.com/quickcalcs/Grubbs1.cfm). Spearman correlation was used to test the association between selected variables. Since correlation is an effect size, the following descriptions of various values of *r* (Spearman correlation coefficient) were used as a guide to estimate the measured values: |*r*| = 0.00–0.19, no correlation; |*r*| = 0.20–0.39, weak correlation; |*r*| = 0.40–0.59, moderate; |*r*| = 0.60–0.079, strong correlation; |*r*| = 0.80–1.0, very strong correlation. Data were analyzed with GraphPad Prism software version 5.01 (GraphPad, La Jolla, CA, USA).

Statistical analysis of the serum metabolome detection was performed with MetaboAnalyst 4.0 [http://www.metaboanalyst.ca; (27)]. Row-wise normalization was performed with a pooled sample from the control group, and column-wise normalization was performed by log2 transformation of the data. Univariate data analysis was performed with a volcano plot with a fold-change threshold of two and with *t*-tests at a threshold of 0.05, as well as with the Mann-Whitney *U*-test. Multivariate data analysis was performed with partial least squares discriminant analysis (PLS-DA) and heat map analysis (Top 25) for visualizing the metabolic differences between FGR and control dams.

## Results

### The hsFLT1/rtTA Mouse Model Led to Ubiquitous Overexpression of hsFLT1

We used transgenic hsFLT1/rtTA mice, in which hsFLT1 can be ubiquitously induced at several time points during pregnancy and is expressed in dams and fetuses or only in dams (depending on the fetal *rtTA* genotype, as described in the mating scheme) (Figure 1B).

**Figure 2:**
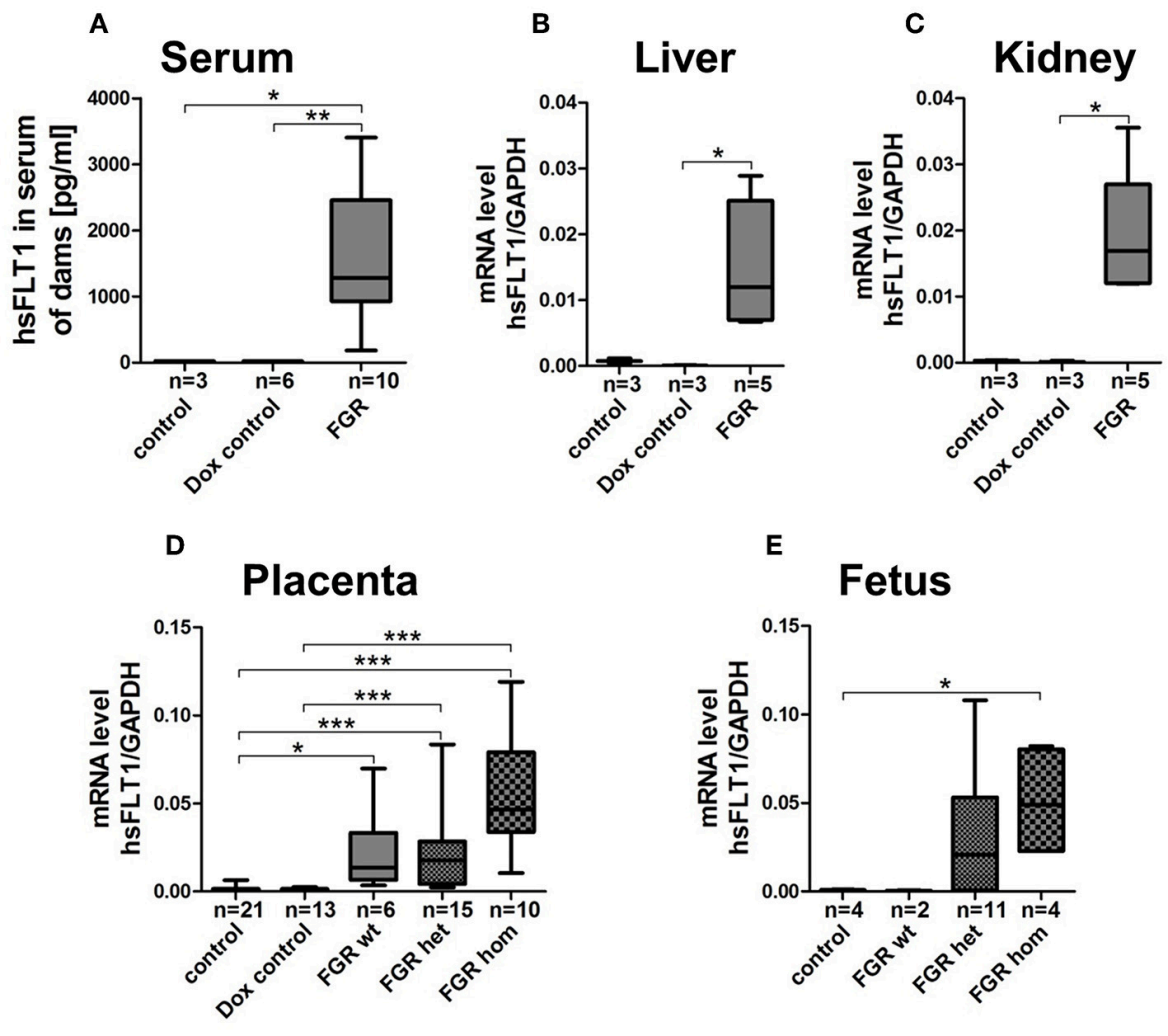
Expression level of hsFLT1 protein in maternal blood serum as measured by ELISA **(A)** and expression of hsFLT1 mRNA in maternal liver **(B)** and kidney **(C)** as well as in placentas **(D)** and fetuses **(E)** as determined by qPCR of the hsFLT1/rtTA mouse model. Expression of human soluble fms-like tyrosine kinase 1 (hsFLT1) occurred only in the mice that exhibited induced hsFLT1/rtTA (reverse tetracycline-controlled transactivator) after treatment with doxycycline (+Dox) (FGR group) and that also exhibited higher placental and fetal *hsFLT1* expression depending on the fetal *rtTA* genotype. Low *hsFLT1* expression was detectable in FGR wild-type (wt) placentas and no *hsFLT1* expression in FGR wt fetuses or in the control and Dox control groups. Samples were obtained from complete placentas and fetuses and from maternal livers and kidneys at day 18.5 post coitum (dpc). Measured mRNA levels were normalized to *Gapdh*. *p < 0.05, **p < 0.01 and ***p < 0.001, as determined by the Kruskal–Wallis test and Dunn's *post hoc* test.

At 18.5 dpc, hsFLT1 was detected in the hsFLT1/rtTA dams treated with Dox at early or midgestation (7.5/10.5 dpc; FGR group) but not in the two control groups, with the following hsFLT1 serum levels: FGR, 1587 ± 294 pg/ml compared to <22 pg/ml for control (*p* < 0.05) and dox control group (*p* < 0.01) (Figure 2A). Also, high levels of *hsFLT1* mRNA were found in liver and kidney tissue from the FGR dams, indicating ubiquitous maternal hsFLT1 overexpression (Figures 2B,C). In addition, elevated *hsFLT1* transcript levels were detected in FGR placentas (FGR wt, het and hom). The placental *hsFLT1* transcript expression strength was associated with the fetal *rtTA* genotype (Figure 2D). The highest level of *hsFLT1* expression was detected in the FGR hom placentas, with lower levels in FGR het and wt placentas and no expression in placentas from either control group or Dox control group. The same held true for FGR hom and FGR het fetuses, because they possess both components of the Tet-On system (the r*tTA* and *hsFLT1* allele) that is necessary for hsFLT1 induction (Figure 2E). In this case, hsFLT1 could be induced by Dox transfer via umbilical cord blood. The FGR wt placentas exhibited only a weak hsFLT1 expression due to the maternal part of the placenta (Figure 2D) and FGR wt fetuses (Figure 2E) exhibited no *hsFLT1* expression because they lack the *rtTA* gene.

### Induction of hsFLT1 in hsFLT1/rtTA Pregnant Mice Resulted in FGR

**Figure 3:**
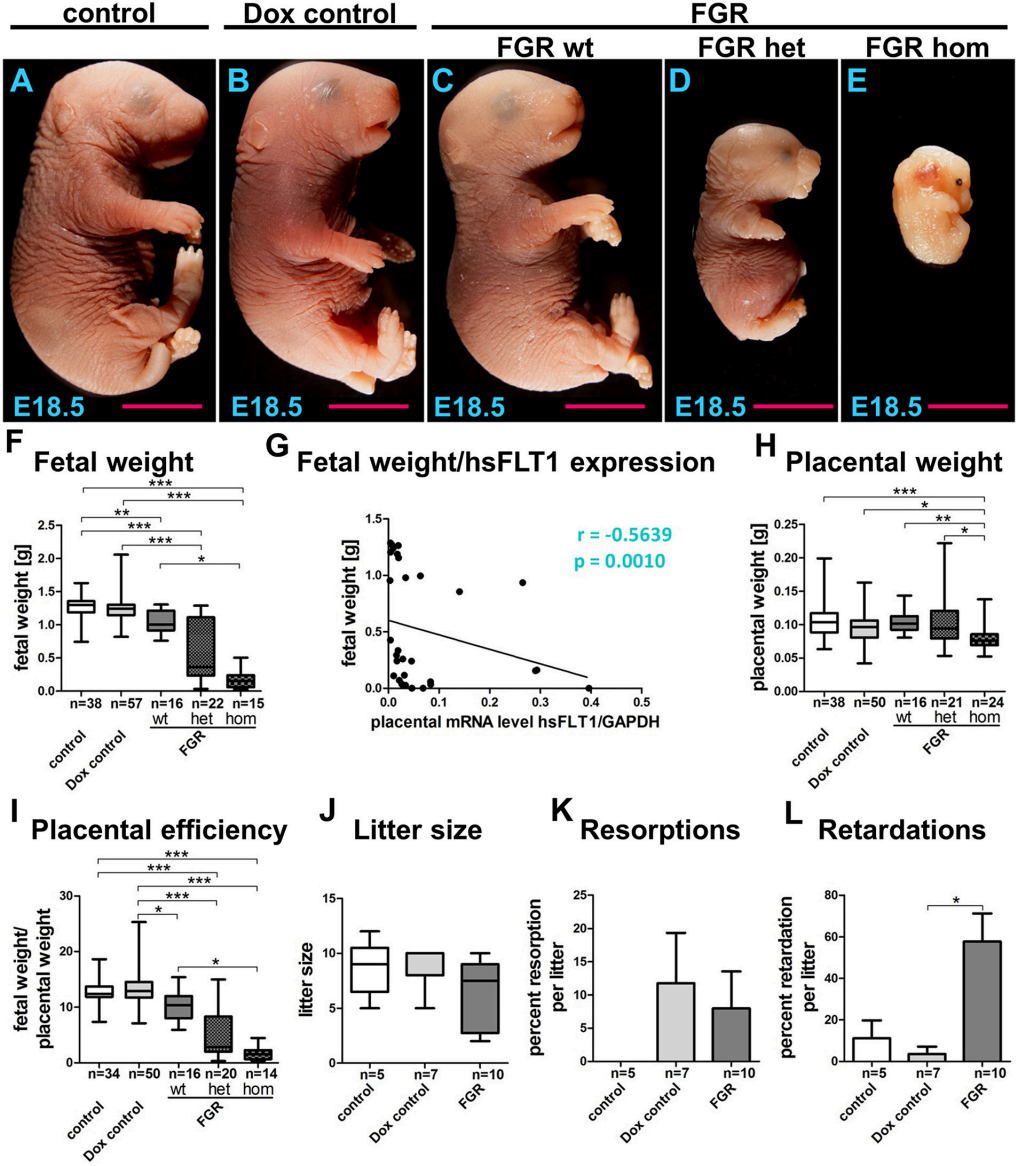
Phenotype of the hsFLT1/rtTA mouse model. Analysis of fetal outcome (fetal weight, litter size, resorptions, and retardations) and the placental phenotype (placental weight, placental efficiency) at day 18.5 post coitum. **(A–E)** Consequences of ubiquitous maternal and/or fetal overexpression of human soluble fms-like tyrosine kinase 1 (hsFLT1) in fetal growth restriction (FGR) homozygous (hom), FGR heterozygous (het), or FGR wild-type (wt) fetuses, showing strong **(A)**, medium **(B)**, or mild **(C)** effects on fetal size in contrast to the control group, which did express hsFLT1 **(D)**. Treating the dam with doxycycline (Dox) (single hsFLT1 mice) produced no negative effects on fetal size **(E)**. These observations were confirmed by analysis of fetal weights **(F)** and by the correlation of fetal weight to placental hsFLT1 expression **(G)**. Placental weight was decreased only in the FGR hom group upon the highest expression of hsFLT1 in fetus and dam **(H)**, whereas placental efficiency (fetal weight/placental weight) is reduced in each FGR group in association with the corresponding hsFLT1 expression levels **(I)**. Litter size **(J)** and number of resorptions **(K)** did not vary between mouse cohorts; however, signs of retardation, such as cyanosis or demise of the fetus, increased in frequency upon hsFLT1 expression **(L)**. Data show hsFLT1/rtTA (FGR) or single hsFLT1 (Dox control) mice treated with Dox at early to midgestation and untreated controls. *p < 0.05, **p < 0.01, ***p < 0.001 as determined by the Kruskal–Wallis test with Dunn's *post hoc* test. Correlation analysis was performed with Spearman's rank correlation coefficient.

Since we found no obvious differences in inducing hsFLT1 expression between treating with Dox at 7.5 dpc (at ectoplacental cone formation) or at 10.5 dpc (at the beginning of placental differentiation; Figure S1), two important reproductive stages in placental development, we present here the combined data collected at both time points, defined as early to midgestation (Figures 2, 3).

Using the hsFLT1/rtTA mouse model, we evaluated fetal and placental weight, litter size per dam, amount of resorption and growth retardation, and placental efficiency (fetal to placental weight) (Figure 3). Maternal overexpression of hsFLT1 with or without fetal overexpression produced various effects on fetal size, depending on the fetal *rtTA* genotype and, therefore, on fetal hsFLT1 expression, categorized as strong (FGR hom; Figure 3E), medium (FGR het; Figure 3D; both maternal and fetal hsFLT1-overexpression), or mild (FGR wt; Figure 3C; maternal hsFLT1 overexpression only). In contrast, fetal size was normal in the control groups that did not express hsFLT1 (Figures 3A,B). Thus, treating the dam with Dox (single hsFLT1 mice) exerted no negative effects on fetal size. These observations were confirmed by analysis of fetal weights (Figure 3F).

It is shown by us that in a dietary mouse model sex-specific placental differences occurred (28). In the current study, the influence of hsFLT1 on fetal outcome seemed to be sex-specific, with a stronger impact on female fetuses than on male fetuses (Figure S2A). However, in the FGR hom and FGR wt groups, fewer male fetuses could be weighed and this difference in numbers may have influenced the ratio between male and female weight outcome (Figure S2C). Statistical analysis found a moderate negative correlation between fetal body weight and placental hsFLT1 expression (*r* = −0.5639; *p* = 0.0010) (Figure 3G). Also, we found a strong correlation between reduced fetal weights and increased fetal hsFLT1 expression (*r* = −0.6691; *p* = 0.0033) (Figure S3A).

Placental weight was decreased in the FGR hom group only at the highest levels of hsFLT1 expression in fetus and dam (Figure 3H). In contrast, placental efficiency (fetal weight/placental weight) was reduced in each FGR group in association with the corresponding hsFLT1 expression levels: we found a weak negative correlation between placental efficiency and placental hsFLT1 expression (*r* = −0.2439; *p* = 0.24) and a moderate negative correlation between placental weight and fetal hsFLT1 expression (*r* = −0.4059; *p* = 0.1188) (Figure 3I; Figures S3B,C). In addition, reduced placental efficiency affects female fetuses more frequently than male ones, as seen before in the differences in fetal weights (Figure S2B). Litter size (Figure 3J) and resorption (Figure 3K) did not vary between the experimental groups; however, Dox treatment seemed to be associated with smaller litter size upon hsFLT1 expression and with a larger number of resorptions, independent of hsFLT1 expression. Signs of retardation, such as cyanosis were frequently observed in the litters of all hsFLT1–expressing dams (FGR group) (Figure 3L).

### Elevated hsFLT1 Levels Led to Severe Changes in Maternal and Fetal Vascularization

**Figure 4:**
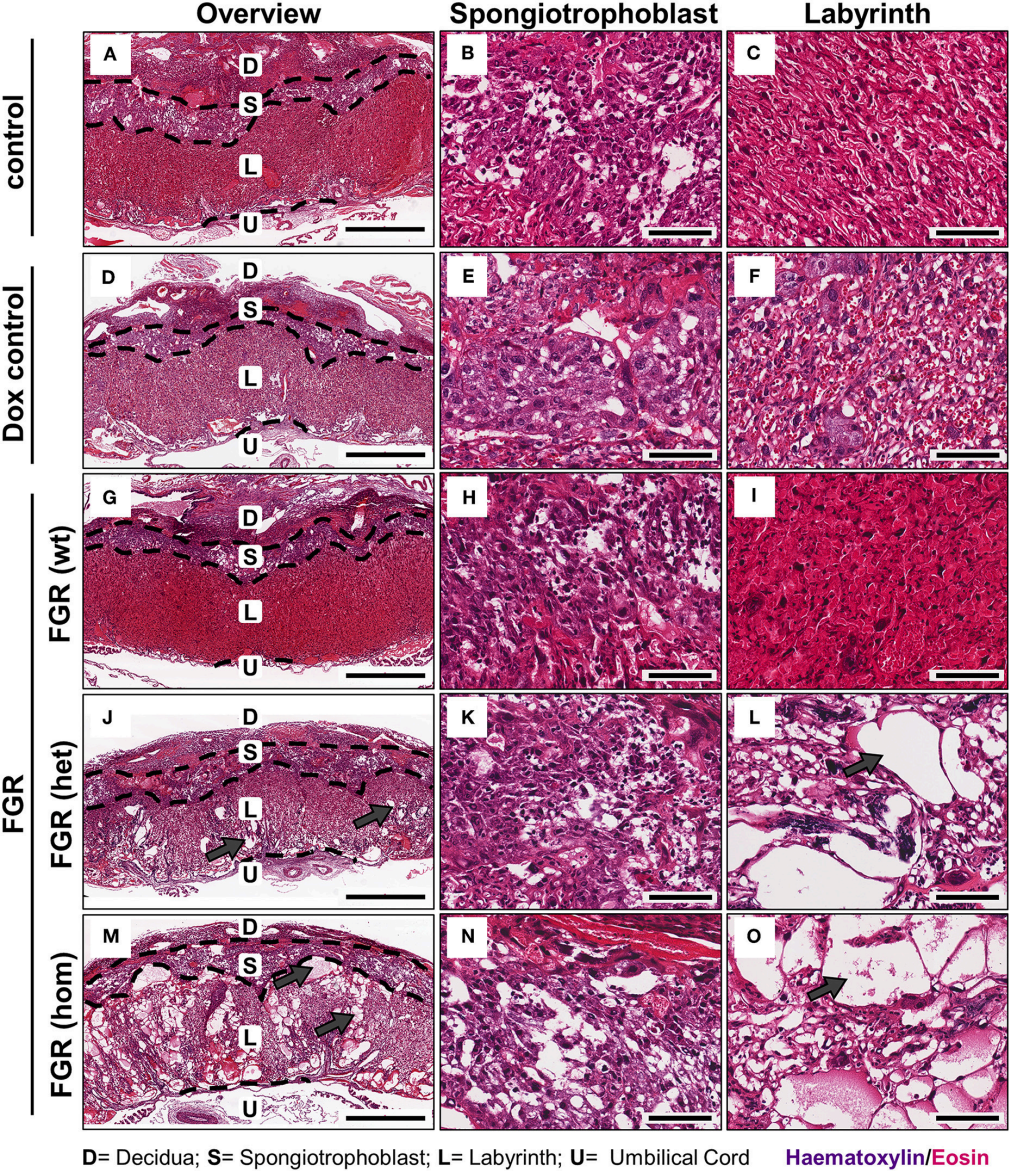
Placental morphology at day 18.5 post coitum in the hsFLT1/rtTA mouse model. Placentas from the various experimental groups were collected and stained with hematoxylin and eosin (H&E). The following groups are shown either in 2× overview (left) or in a 20× detailed structure view of spongiotrophoblast (middle) and labyrinth (right): control [*n* = 16 **(A–C)**] and doxycycline (Dox) control placentas [*n* = 12 **(D–F)**], fetal growth restriction wild-type [FGR wt; *n* = 9 **(G–I)**], FGR heterozygous het; *n* = 3 **(J–L)** and FGR homozygous placentas [hom; *n* = 10 **(M–O)**]. High-expressing human soluble fms-like tyrosine kinase 1 (hsFLT1) FGR hom and FGR het placentas (maternal and placental hsFLT1 expression) exhibited enlarged blood-filled spaces (lacunas, indicated by gray arrows) within the entire placenta, but low-expressing hsFLT1 FGR wt placentas (with maternal hsFLT1 expression only), non-expressing hsFLT1 control placentas, and Dox control placentas did not exhibit such lacunas [**(A,D)** compared to **(G,J,M)**]. The lacunas were located not only in the spongiotrophoblast [**(B,E)** compared to **(H,K,N)**], but also in the labyrinth of hsFLT1–expressing placentas [**(C,F)** compared to **(I,L,O)**]. Interestingly, the FGR wt placentas exhibited a phenotype with a morphology between those of FGR hom/het placentas and control placentas, in which the labyrinth compartment is more densely characterized by an intense staining pattern. Scale bar 2× overview = 1,000 μm; 20× details = 100 μm. D, decidua; L, labyrinth; S, spongiotrophoblast; U, umbilical cord.

**Figure 5:**
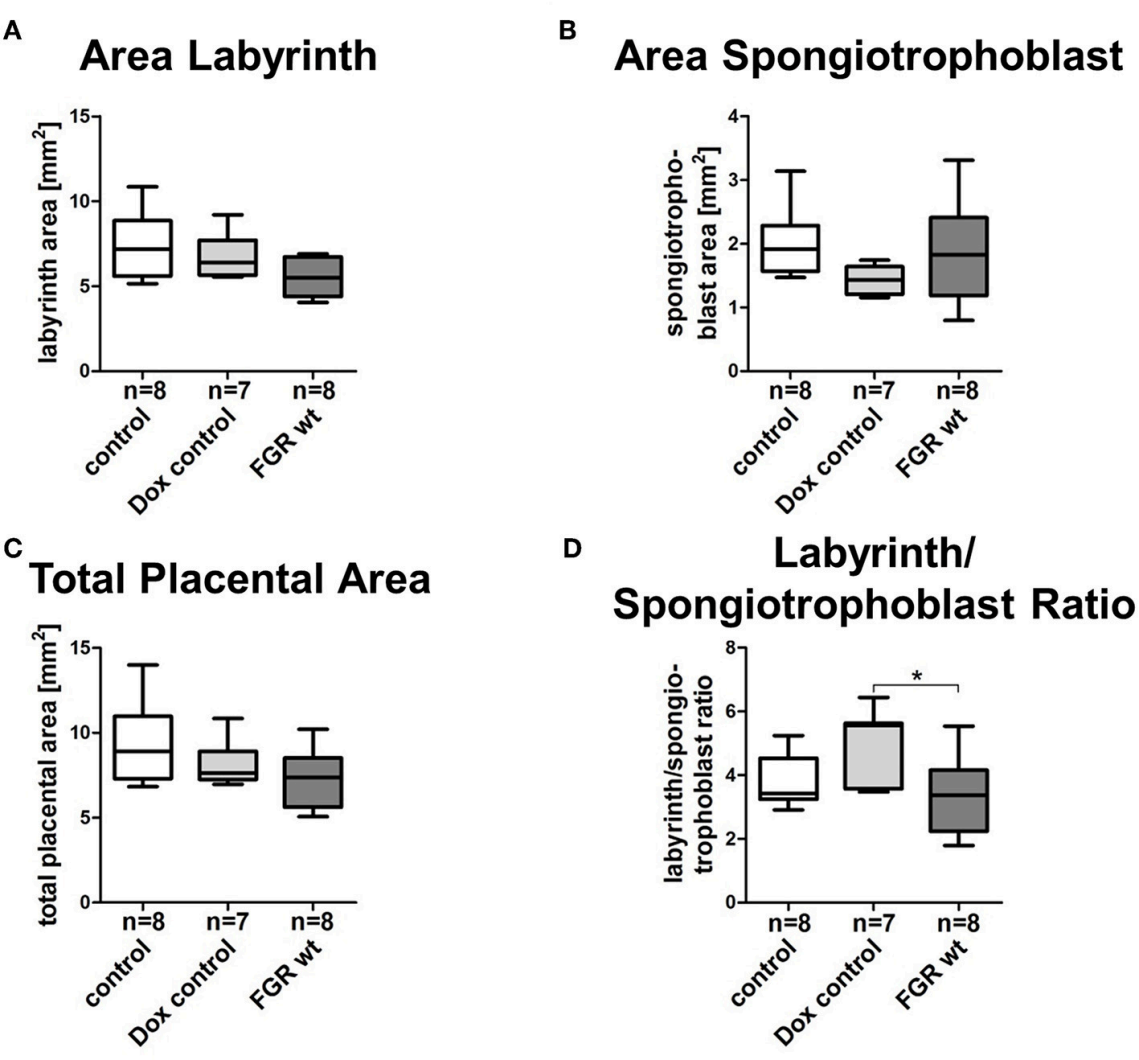
Morphometric analysis of placentas in the hsFLT1/rtTA mouse model. Analysis of the labyrinthine **(A)**, spongiotrophoblast **(B)**, and total placental area **(C)** as well as labyrinth to spongiotrophoblast area ratio **(D)** of human soluble fms-like tyrosine kinase 1 (hsFLT1) expressing fetal growth restriction wild-type placentas (FGR wt; *n* = 8), compared to control (*n* = 8), and doxycycline (Dox) control (*n* = 7) placentas. FGR wt placentas showed slight reduction in labyrinth and total placental area and labyrinth to spongiotrophoblast ratio compared to both control groups, whereas spongiotrophoblast area was slightly increased. Data is presented in box and whisker plot. *p < 0.05 as determined by the Kruskal–Wallis test with Dunn's *post hoc* test.

FGR is often associated with placental dysfunction and malnutrition (1). Hence, we asked whether the anti-angiogenic factor hsFLT1 affects placental development, vascularization, and function. Histologic analysis of the hsFLT1–expressing placentas in the FGR hom and het groups showed a severe impairment in placental structure due to extremely enlarged blood-filled spaces, called lacunas (Figures 4J–O); this impairment did not appear in control placentas (Figures 4A–F). The largest alterations in placental morphology were found in the FGR hom group and were associated with high placental hsFLT1 levels. The large lacunas found in the FGR hom and het placentas appeared to sprout from the chorionic plate into the labyrinth, which is responsible for nutrient exchange (Figures 4K,L,N,O). The FGR wt placentas, which are characterized by a weak expression of hsFLT1 due to the maternal part, exhibited a morphological phenotype different from that of FGR hom/het placentas and control placentas, in which the labyrinth compartment was appropriately differentiated, with branched villi containing fetal blood vessels and trophoblast layers lining the longitudinally arranged maternal sinusoids (Figures 4G–I). To analyze this more deeply we assessed FGR wt placentas for total placental area as well as labyrinth and spongiotrophoblast layer (Figure 5). Total placental area (Figure 5C) and labyrinth area (Figure 5A) tended to be slightly decreased in the FGR wt placentas in comparison to controls and in addition the labyrinth to spongiotrophoblast ratio (Figure 5D) was also reduced in FGR wt placentas compared to controls.

**Figure 6:**
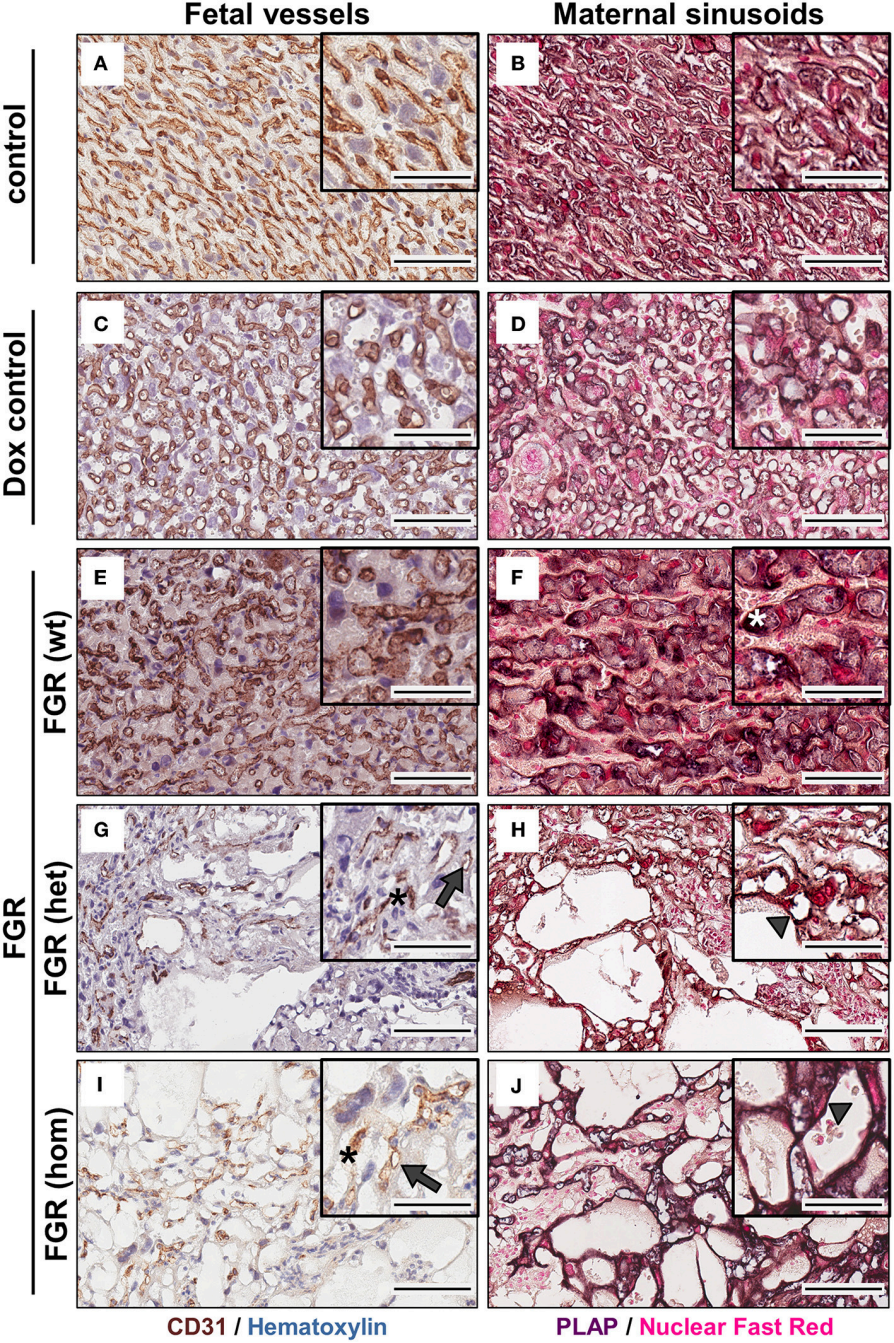
Analysis of fetal (left) and maternal (right) vascularization in the placental labyrinth in the hsFLT1/rtTA mouse model. Immunohistochemical staining of Cd31 (brown staining) in the labyrinth area indicates fewer fetal vessels (indicated by gray arrows) and inadequate formation of blood spaces (indicated by black asterisks) in high-expressing human soluble fms-like tyrosine kinase 1 (hsFLT1) fetal growth restriction homozygous (FGR hom; *n* = 10) **(I)** and FGR heterozygous (het; *n* = 3) **(G)** placentas (maternal and fetal hsFLT1 expression) than in low-expressing hsFLT1 FGR wt [*n* = 9 **(E)**] (maternal expression only) and non-expressing hsFLT1 control (*n* = 16) and doxycycline (Dox) control (*n* = 12) placentas **(A,C)**. For Cd31 staining nuclei are counterstained in blue. Cells lining dilated vessels (lacunas) in high-expressing hsFLT1 FGR hom [*n* = 10 **(J)**] and FGR het [*n* = 3 **(H)**] placentas exhibited placental alkaline phosphatase (PLAP) activity (dark purple staining), a finding indicating the presence of sinusoidal trophoblast giant cells (S-TGCs); therefore, these vessels are characterized as maternal sinusoids (indicated by gray arrowheads). In addition, PLAP-positive vessels in low-expressing hsFLT1 FGR wt placentas [*n* = 9 **(F)**] exhibited a different phenotype, especially for the maternal sinusoids, with more longitudinally arranged and slightly larger sinusoids than in the other groups (indicated by white asterisks). In contrast, the controls [control *n* = 16 **(B)** and Dox control *n* = 12 **(D)**] did not exhibit dilatation of maternal sinusoids. For PLAP staining nuclei are counterstained in light red. Scale bar 20× details = 100 μm and 40× details = 50 μm.

**Figure 7:**
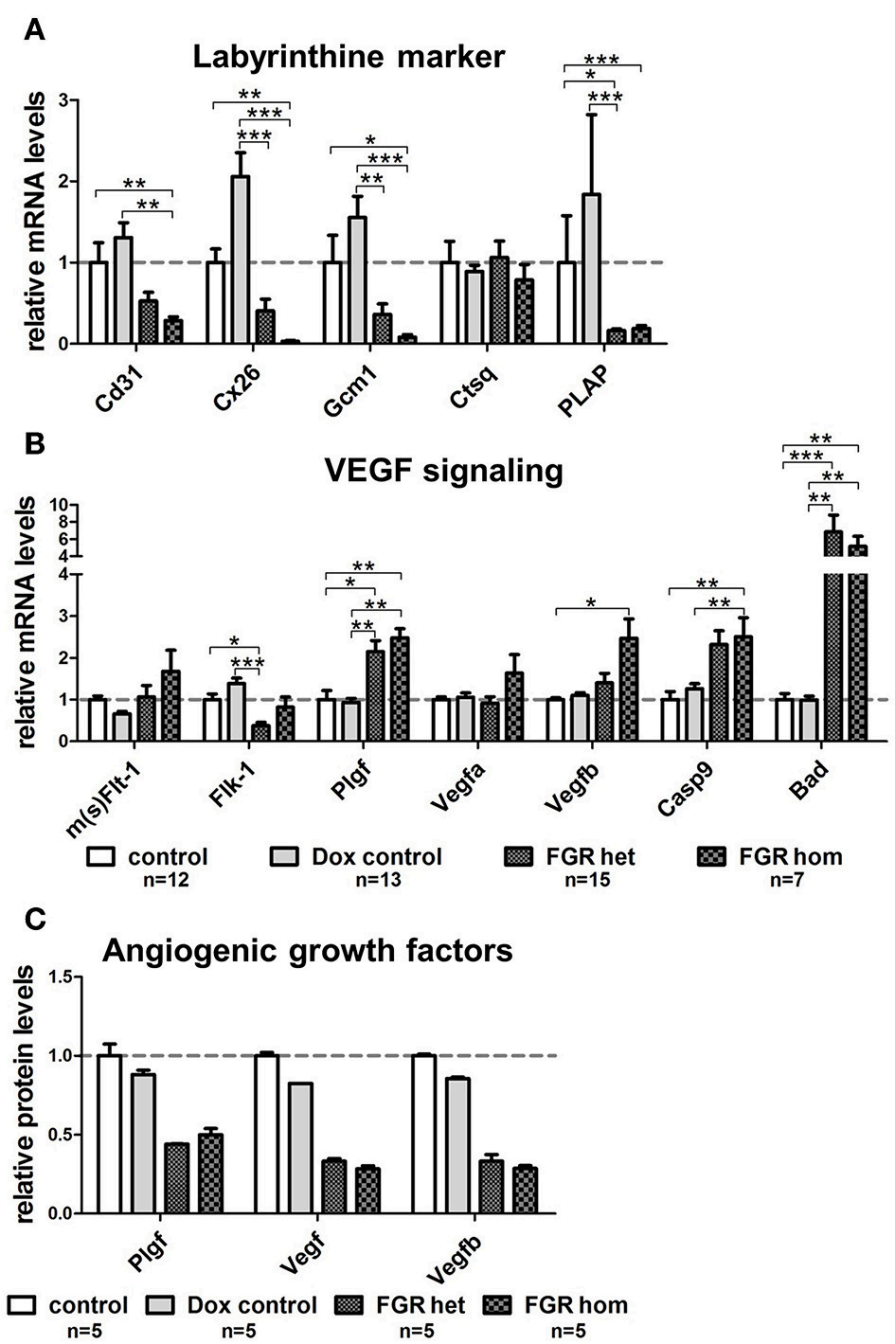
Gene expression analysis of important placental labyrinthine markers and Vegf signaling molecules in the hsFLT1/rtTA mouse model. Quantitative reverse transcription polymerase chain reaction (qRT-PCR) of marker genes in placentas of each group: fetal growth restriction homozygous (FGR hom; *n* = 7), FGR heterozygous (het; *n* = 15), control (*n* = 12), and doxycycline (Dox) control (*n* = 13). **(A)** The mRNA level of fetal endothelial cell marker *Cd31* was lower in placentas expressing human soluble fms-like tyrosine kinase 1 (hsFLT1) (FGR hom; FGR het) than in control placentas (control and Dox control). In addition, the expression of syncytiotrophoblast markers, such as gap junction protein connexin 26 (*Cx26*) and differentiation-promoting transcription factor glial cell missing one (*Gcm1*), is lower in hsFLT1–expressing placentas (FGR hom more pronounced than FGR het) than in control placentas (control and Dox control). The expression of maternal sinusoidal trophoblast giant cell markers, such as placental alkaline phosphatase (*PLAP*), was also lower in hsFLT1–expressing placentas than in control placentas, whereas cathepsin Q (*Ctsq*) expression seemed to be unaffected by hsFLT1 expression in all groups. **(B)** Murine mRNA levels of *sFlt-1* and *Flt-1* (*m(s)Flt-1*) exhibited no clear up- or downregulation between groups (no discrimination between murine *sFlt-1* and murine *Flt-1* possible at the mRNA level), whereas the expression of murine fetal liver kinase 1 (*Flk-1*) was lower in FGR hom and FGR het placentas than in control placentas (control and Dox control). Growth factors, such as placental growth factor (*Plgf*; mainly binding to sFlt-1 and Flt-1), vascular endothelial growth factor A (*Vefga*; mainly binding to sFlt-1, Flt-1, and Flk-1), and *Vegfb* (mainly binding to sFlt-1 and Flt-1), are upregulated at the time of hsFLT1 expression (FGR hom and FGR het), a finding correlating with increasing levels of hsFLT1. Nevertheless, the proapoptotic markers caspase9 (*Casp9*) and Bcl-2–associated death promoter (*Bad*) downstream of Flk-1 are more highly upregulated upon hsFLT1 expression in FGR hom and FGR het placentas than in either control group (control and Dox control). Samples were obtained from complete placentas at day 18.5 post coitum (dpc). Measured mRNA levels were normalized to glyceraldehyde-3-phosphate dehydrogenase (*Gapdh;* except for *Casp9* and *Bad*, which were normalized to *Flk-1*), and control group levels were set at 100% (dotted line). Data is presented as mean ± standard error of the mean. *p < 0.05, **p < 0.01, and ***p < 0.001 determined by the Kruskal–Wallis test with Dunn's *post hoc* test. **(C)** Protein levels of Plgf, total Vegf, and Vegfb were reduced upon hsFLT1 expression in FGR hom (*n* = 5) and het (*n* = 5) placentas compared to control (*n* = 5) and Dox control group (*n* = 5). Data is presented as mean ± standard deviation.

To determine whether the dilated vessels in the FGR het and hom placentas were of maternal or fetal origin, we immunostained paraffin sections for the fetal endothelial cell (EC) marker cluster of differentiation 31 (Cd31; Figures 6A,C,E,G,I). Cd31 staining showed that the number of fetal vessels was lower in the hsFLT1–expressing placentas (FGR hom and het) (Figures 6G,I) than in control placentas (Figures 6A,C), with the strongest reduction in the FGR hom placentas. Moreover, in FGR hom and FGR het placentas, single ECs were detected in the labyrinthine compartment, but they did not form appropriate fetal capillaries (Figures 6G,I). The Cd31 signal in the stained FGR wt placentas (Figure 6E) was similar to that in the control placentas (Figures 6A,B). We also found lower levels of *Cd31* in hsFLT1–expressing FGR hom and FGR het placentas than in control placentas at the transcript level (Figure 7A).

Staining for PLAP, which is exclusively present in the sinusoidal trophoblast giant cell (S-TGC) subtype and which lines the maternal sinusoids in the labyrinth (Figures 6B,D,F,H,J), demonstrated that the observed large lacunas in the hsFLT1–expressing placentas (FGR hom and FGR het) were maternal sinusoids (Figures 6H,J). The FGR wt placentas exhibited maternal sinusoids that were more longitudinally arranged and slightly larger or more swollen (Figure 6F) than those exhibited by the other groups. All maternal sinusoids in the control placentas exhibited a normal phenotype (Figures 6B,D). *PLAP* mRNA expression was also lower in hsFLT1–expressing placentas (FGR hom and het) than in control placentas (Figure 7A), whereas the levels of an additional marker of S-TGCs, cathepsin Q (*Ctsq*), were only moderately reduced in FGR hom placentas and were unchanged in the experimental groups (Figure 7A).

### Elevated hsFLT1 Levels Led to Severe Changes in Placental Differentiation in the Labyrinthine Compartment, as Characterized by Inhibition of Vegf Signaling

Because we found that hsFLT1 expression exerted a strong effect on changes in the placental labyrinthine compartment, we also quantified syncytiotrophoblast differentiation marker genes of the labyrinth, such as the leading transcription factor glial cell missing 1 (*Gcm1*) and the gap junction protein connexin 26 (*Cx26*), which is located between the two syncytiotrophoblast layers (Figure 7A). We found lower transcript levels of both differentiation marker genes (*Gcm1* and *Cx26*) in the FGR hom and het placentas than in control placentas, corresponding to the hsFLT1 levels in each experimental group. We also analyzed various markers on transcript level for spongiotrophoblast and glycogen cells, which are mainly involved in endocrine regulation. These markers include the prolactin *Prl3d1* as a marker for parietal trophoblast giant cells (P-TGCs), *Cx31*, and trophoblast-specific protein alpha (*Tpbpa*), all of which are upregulated upon hsFLT1 expression (refer to Figure S4A).

We also investigated transcriptional changes in the endogenous Vegf signaling cascade in the placentas of the various experimental groups. We analyzed most of the relevant genes in this pathway, such as the murine/intrinsic variants of *sFlt-1*/*Flt-1* (*m(s)Flt-1*); fetal liver kinase 1 (*Flk-1*); the ligands *Plgf*, *Vegfa*, and *Vegfb*; and the proapoptotic markers downstream of Flk-1, such as caspase 9 (*Casp9*) and Bcl-2–associated death promoter (*Bad*) [reviewed by Koch and Claesson-Welsh (29)] (Figure 7B). The mRNA levels of murine *sFlt-1*/*Flt-1* and its ligands *Plgf*, *Vegfa*, and *Vegfb* were higher in the FGR group, primarily in the FGR hom group, than in the control groups. In contrast, the levels of *Flk-1*, which is located on fetal ECs (30), are lower in hsFLT1–expressing placentas (FGR hom and FGR het) than in control placentas. In contrast, other Vegf receptors and Vegf isoforms, such as Fms-related tyrosine kinase 4 (*Flt-4*), *Vegfc*, and *Vegfd*, were not regulated by hsFLT1 overexpression (Figure S4A).

To analyze the growth factors of the Vegf signaling cascade also on protein level we used a Proteome Profiler™ Mouse Angiogenesis Antibody Array to simultaneously detect 53 angiogenesis-related proteins in a single sample. We found a downregulation of total Vegf, Vegfb in particular, and Plgf in the FGR hom and het placentas compared to the controls (Figure 7C). In addition, the Angiogenesis Antibody Array exhibits an upregulation of the angiogenesis-related proteins tissue factor, Serpin E1, and Serpin F1 upon hsFLT1 expression in FGR hom and het placentas compared to both control groups (Figure S4B).

Furthermore, we found that the proapoptotic molecules *Bad* and *Casp9* are strongly increased upon hsFLT1 upregulation (Figure 7B). Both factors signal downstream of Flk-1 and have been shown to be strongly negatively regulated by Vegfa (29). hsFLT1–expressing FGR hom and het placentas exhibited increased transcript levels of both proapoptotic markers.

Overall, we found that, upon hsFLT1 expression, 22 of 34 genes were differently regulated in FGR groups than in control groups, and these differences led to distinct transcriptomic profiles (Figure S4A).

### Elevated hsFLT1 Levels Altered the Placental Transporter System and Increased the Total Levels of Phosphatidylcholine in Maternal Serum From hsFLT1/rtTA Mice

**Figure 8:**
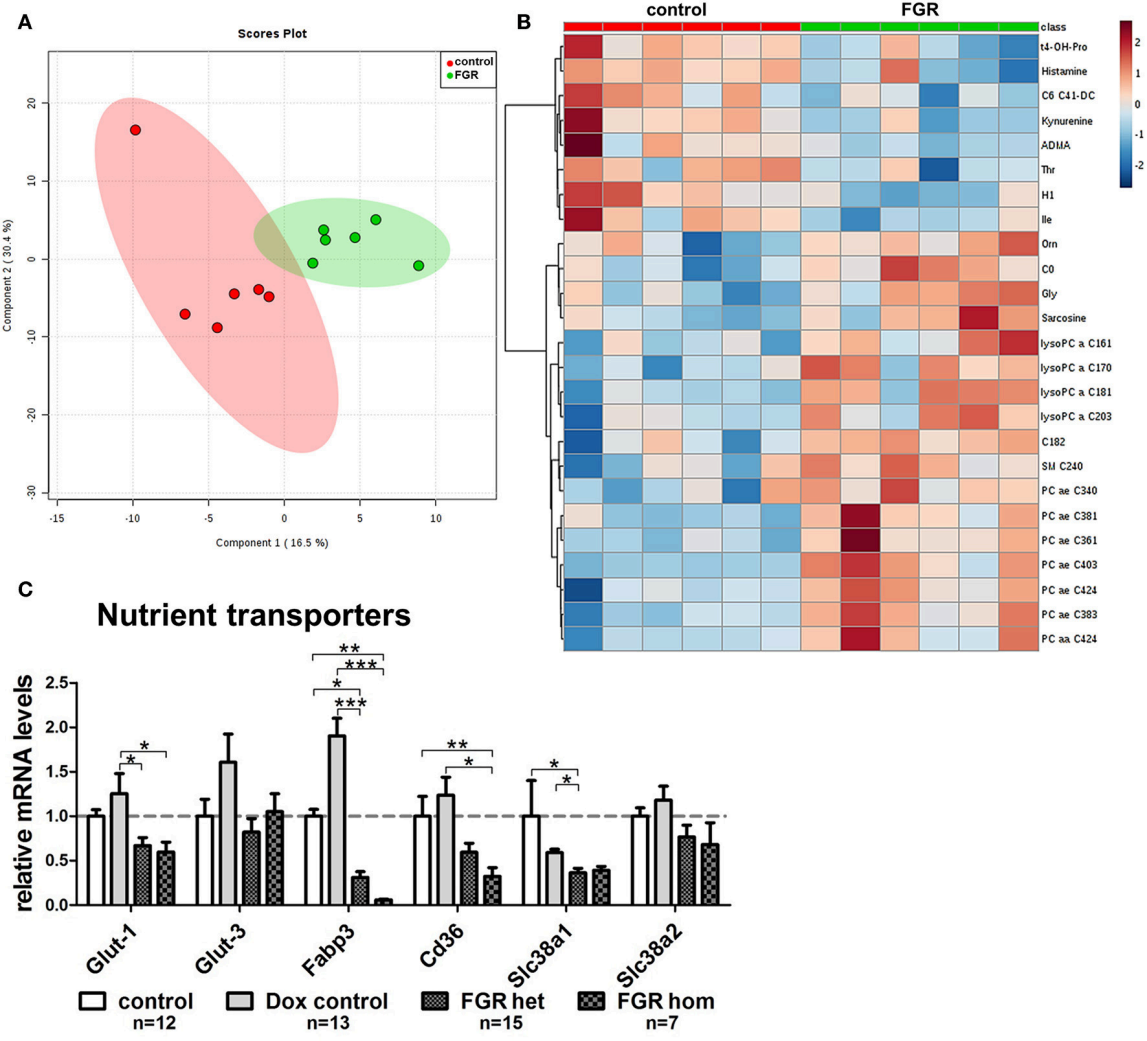
Maternal metabolome analysis and placental expression of nutrient transporter genes in the hsFLT1/rtTA mouse model. **(A)** Supervised partial least squares discriminant analysis (PLS-DA) of 152 metabolites detected the main metabolomic differences in serum from fetal growth restriction dams (FGR; *n* = 6; green dots) and control dams (*n* = 6; red dots). **(B)** Heat map representation of the top 25 modified metabolites in each group (*n* = 6 each) mainly indicated accumulation of lysophosphatidylcholines and phosphatidylcholines in serum from hsFLT1–expressing dams (color-coding intensity in the red spectrum shows an increase in the number of given metabolites, and color intensity in the blue spectrum shows a decrease in the number of the given metabolites). **(C)** Results of quantitative reverse transcription polymerase chain reaction (qRT-PCR) analysis of nutrient transporters in placentas from each group: FGR homozygous (hom; *n* = 7), FGR heterozygous (het; *n* = 15), control (*n* = 12), and doxycycline (Dox) control (*n* = 13). The mRNA expression levels of glucose transporters *Glut-1* and *Glut-3*, fatty acid transporters fatty acid binding protein 3 (*Fabp3*) and *Cd36*, and amino acid transporters solute carrier family members one and two (*Slc38a1* and *Slc38a2*) are lower in hsFLT1–expressing placentas (FGR hom and FGR het) than in control placentas (control and Dox control); the strongest decrease was observed in the fatty acid transporters. Samples were obtained from complete placentas at day 18.5 post coitum (dpc). Measured mRNA levels were normalized to glyceraldehyde-3-phosphate dehydrogenase (*Gapdh*), and the control group was set at 100% (dotted line). Data is presented as mean ± standard error of the mean. *p < 0.05, **p < 0.01, ***p < 0.001 as determined by the Kruskal–Wallis test with Dunn's *post-hoc* test.

The expression level of important nutrient transporter genes for the transport of glucose, amino acid, and fatty acid across the placental barrier was screened. For each transport pathway, we analyzed two transporters: for glucose transport, glucose transporter 1 (*Glut-1*; localized in syncytiotrophoblast (ST) layer I and II [reviewed by Winterhager and Gellhaus (20)] and 3 (*Glut-3*; localized in ST layer I); for fatty acid transport, the fatty acid translocase *Cd36* and fatty acid binding protein 3 (*Fabp3*) (both ST layer I and II); and for amino acid transport, solute carrier family 38, members one and two (*Slc38a1* in ST layer I and II); *Slc38a2* only in ST layer II). The expression of all examined transporters was lower in mice expressing induced hsFLT1 (FGR hom and FGR het) than in uninduced controls, with the strongest decrease in the expression of fatty acid transporters (Figure 8C).

Since we observed a strongly impaired placental morphology and changes in the transport system, we expected that the induced hsFLT1/rtTA dams (FGR group) and uninduced dams (control group) would exhibit unique metabolomic profiles. Using the Biocrates AbsoluteIDQ® p180 Kit, we investigated 188 endogenous metabolites from five compound classes by tandem mass spectrometry (MS/MS). Metabolites whose levels were below the lower limit of quantification (<LLOQ) were excluded; the remaining 152 metabolites were included in the analysis. To visualize the main metabolic differences between experimental groups, we performed multivariate data analysis with partial least squares discriminant analysis (PLS-DA) and heat map analysis (the 25 top changed metabolites are shown in Figure 8B). PLS-DA showed a clear distinction in the variable importance of projection (VIP) scores between the two experimental groups (FGR vs. controls) (Figure 8A). A heat map representation of the top 25 modified metabolites showed distinct metabolic footprints between the FGR group and the control group, with 17 upregulated and eight downregulated metabolites (Figure 8B). Most of the upregulated metabolites were lipids, whereas most of the downregulated metabolites were amino acids or biogenic amines. Furthermore, volcano plot analysis, a combination of fold change (FC = 2) and *t*-test results (*p* < 0.05), showed that 13 changed metabolite levels were found in serum from dams (FGR and controls). Ten of these were upregulated, and most were long chain fatty acid phosphatidylcholine (PC)/lysophosphatidylcholines or the amino acid glycine (Gly) and its byproduct/precursor sarcosine (Figure 8B; Table 3). All three downregulated metabolites were biogenic amines.

**Table 3:** Top 13 regulated metabolites in serum among dams in the hsFLT1/rtTA mouse model.

	**Fold change**	**log2(FC)**	***p*–value**	**-log10(p)**
PC ae C40:3	0.27987	−1.8372	0.001	3.1843
PC ae C38:3	0.45671	−1.1306	0.002	2.7442
lysoPC a C18:1	0.449	−1.1552	0.004	2.4119
PC ae C38:1	0.36779	−1.443	0.005	2.2804
Gly	0.35951	−1.4759	0.009	2.0239
PC ae C42:4	0.32027	−1.6427	0.010	2.0139
PC aa C42:4	0.3671	−1.4458	0.012	1.909
Sarcosine	0.44167	−1.1789	0.019	1.7266
lysoPC a C20:3	0.31089	−1.6855	0.024	1.6226
PC aa C40:3	0.40968	−1.2874	0.041	1.3855
Kynurenine	2.1765	1.122	0.010	2.0002
t4-OH-Pro	2.0757	1.0536	0.013	1.9025
Histamine	2.0072	1.0052	0.021	1.6826

*aa, diacyl; ae, acyl-alkyl; FC, fold change; Gly, glycine; lysoPC, lyso phosphatidylcholine; p, probability; PC, phosphatidylcholine; t4-OH-Pro; trans-4-Hydroxyproline*.

### Overexpression of hsFLT1 Resulted in Only Small Epigenetic Changes in the Placentas

We have previously shown that overexpression of placenta-specific sFLT1 by lentiviral gene delivery results in small changes in DNA methylation (19). Using the previous lentiviral model, we found DNA methylation of *Igf2-DMR2, H19-ICR*, and *LINE1*. Two of the five CpG positions in the *Igf2* gene were hypomethylated, but there was no change in general DNA methylation.

Consequently, in the current study we measured DNA methylation at the same loci: *Igf2-DMR2* (five positions), *H19-ICR* (three positions), and *LINE1* (five positions) (Figure S5). However, in the FGR wt placentas we found changes in DNA methylation of *LINE1* at CpG position three, but no further changes in DNA methylation at the other four CpG positions. In addition, no changes in DNA methylation were found for *H19-ICR* and *Igf2-DMR2*.

## Discussion

Various sFLT1 mouse models based on adenoviral transduction (31, 32) have been used in FGR/PE research; most of these models exhibited an accumulation of sFLT1 in the liver. The effects of sFLT1 on the maternal endothelium and the maternal organs have been described [reviewed by Lecarpentier and Tsatsaris (17)]. However, less is known about the direct and indirect influence of sFLT1 on placental development and function. Therefore, we generated transgenic inducible hsFLT1/rtTA mice, in which hsFLT1 expression can be ubiquitously induced in dams and fetuses by Dox administration at chosen time points between early gestation and midgestation during pregnancy, as in humans.

Since Dox crosses the placental barrier, hsFLT1/rtTA fetuses can express hsFLT1 just as their dams do. Because of the limitations of inheritance rules, we could not generate pregnancies in these transgenic hsFLT1/rtTA mice during which the dams overexpressed hsFLT1 and the fetuses did not. Therefore, we used hsFLT1/rtTA heterozygous mating to create various levels of hsFLT1 expression in fetuses within a single dam, depending on the fetal *rtTA* genotype (hom, het, or wt). With these three possibilities of hsFLT1 expression, we could discriminate between overexpression of hsFLT1 by dams and by fetuses or placentas and, thus, between the effects of maternal hsFLT1 overexpression and those of fetal/placental hsFLT1 overexpression on both fetus/placenta and mother during pregnancy. The rationale and premise for this hsFLT1 mouse model is that we are able to analyze if hsFLT1—only maternally expressed- also affects the fetus indirectly (by altering mediators) or directly via a transplacental transport of hsFLT1 from the mother to fetal circulation. It would require an active transport mechanism, of hsFLT1 with 120 kDa to cross the placental barrier, because the limit of membrane transfer for proteins via the placenta is believed to be ~500 Da (33). Kumasawa et al. (18) suggested that hsFLT1 in the mother's circulation can pass through the placenta into the fetus and thus contribute to FGR, but evidence was missing. Anyway, we observed only a very small effect in the wt fetuses on fetal growth. Thus, the amount of hsFLT1 levels not only in the maternal but predominantly in the fetal circulation seems to be important. A placental transfer of hs to the fetus is not yet proven by us or others and would need further investigations in future studies. Furthermore, inducing the hsFLT1 expression directly in the fetus itself allows analyzing direct effects of hsFLT1 on placental development and the possible consequences for the dam to develop heart diseases in later life upon fetal expression of hsFLT1.

In addition to the theory that hsFLT1 is transported across the placenta to the fetus it cannot be excluded that the slight growth restriction of FGR wt fetuses is due to the presence of hsFLT1 in the maternal decidua. The maternal sinusoids which exhibit high levels of hsFLT1 bathe the placenta and could influence changes in placental function, as indicated by reduced placental efficiency and FGR. The association between increased sFLT1 levels in maternal circulation and FGR of the fetuses is already shown in humans (34). The results of hsFLT1/rtTA homozygous mating showed that hsFLT1/rtTA homozygous fetuses did not survive after birth, probably because of strong growth retardation or associated other not known malformations. Thus, the survival rate and the reasons for stillbirth in the FGR groups must be confirmed in experiments focused on fetal outcome.

Focusing on the effect of hsFLT1 on placental development, we found that placental weights were reduced in FGR hom placentas but not in FGR het and wt placentas but a decrease in placental efficiency was found in all FGR groups. In the sFLT1 lentiviral mice (19) both, the fetal weight and the placental weight of sFLT1–transduced mice were reduced. These differences in placental weights could be due to the different model systems because in the lentiviral mouse model hsFLT1 is permanently expressed in the trophoblast cells already from the blastocyst stage onwards.

The placental phenotype described here, with enlarged maternal blood sinusoids and reduced numbers of fetal blood vessels, has not been observed in other sFLT1 mouse models (31, 32, 35). The exclusively maternal overexpression of hsFLT1 in the FGR wt group produced results very similar to those found in the placenta-specific lentiviral mice published by Kumasawa et al. (18) and our group (19). Both groups evidenced a reduced labyrinthine layer and in the study of Kumasawa et al. (18) also a reduction in Cd31 expression. The observed alterations in the maternal blood spaces by Kumasawa et al. (18) could not be seen in our lentiviral mouse model (19) but in the present study using the hsFLT1 maternally overexpressing mice.

We induced hsFLT1 expression in early to midgestation, at the leading time point of chorioallantoic attachment followed by fetal vasculature branching to form dense villi within the murine labyrinth (8.5–10.5 dpc), processes that are strictly regulated by members of the Vegf, Plgf, fibroblast growth factor (Fgf), and transforming growth factor beta (Tgf-β) families (36, 37). At this time point of gestation, the inhibition of angiogenesis by overexpression of hsFLT1 could have the strongest effect and strengthens the finding that Vegf is required for placental development following 7.5 dpc. This finding was closely associated with the change in expression patterns of Vegf signaling molecules and apoptotic markers in the FGR hom and het placentas. Since we induced hsFLT1 expression upon 7.5 dpc, hsFLT1 could also have influenced the yolk sac function, which is the main source of nutrition to the fetus before 10.5 dpc of pregnancy (38). Thus, we cannot exclude that impaired development of the yolk sac function also contributes to FGR. However, taken into account that the later induction of hsFLT1 on 10.5 dpc where the yolk sac is already differentiated lead to the same effect on fetal and placental growth, we suggest a minor role of yolk sac dysfunction in our model.

The unraveling of hsFLT1–associated impairment of the Vegf signaling cascade showed a strong upregulation of the endogenous binding partners of murine sFlt-1 on transcript level in the placentas: the murine variants of *Plgf*, *Vegfa*, and *Vegfb* exhibited a kind of counterregulation to the increase in hsFLT1, indicating the binding of hsFLT1 to the murine ligands. This could be strengthen by a reduction of Vegf and Plgf protein levels in the FGR hom and het groups maybe causing an impaired Vegf signaling, indicated by mRNA upregulation of Vefg-related apoptosis markers. Indeed, Szalai et al. (32) found that hsFLT1 can bind and sequester murine Plgf *in vivo*. The mRNA expression of membrane-bound Vegf receptor 2 (*Flk-1*), which is the leading receptor for angiogenesis and is expressed by placental/fetal ECs (30, 39), was lower in FGR hom and het placentas than in control placentas. The reduction of *Flk-1* transcript levels was combined with a reduction in *Cd31* transcript levels and in the number of fetal ECs.

Moreover, the mRNA expression of proapoptotic markers such as *Casp9* and *Bad* downstream of Flk-1 was highly upregulated upon hsFLT1 expression in FGR hom and het placentas, a finding that argues for apoptosis of fetal ECs and the consequent reduction in *Cd31* signals and in the number of fetal ECs. Jiang et al. (40) found that sFLT1 mediates oxidative stress on trophoblast cells during PE and thereby increases apoptosis. We hypothesize that the same holds true for fetal ECs. These findings indicate that placental Vegf signaling is impaired, and this impairment probably inhibits placental vessel development. Therefore, we hypothesize that the observed FGR phenotype in the hsFLT1/rtTA fetuses results mainly from impaired Vegf signaling via Flk-1 in the placenta, which is triggered by excessive signaling of the anti-angiogenic molecule hsFLT1 and, as a consequence, by reduced binding of Vegfa and Vegfb to Flk-1. In contrast, other Vegf receptors and Vegf isoforms, such as *Flt-4, Vegfc*, and *Vegfd*, seem not to be differentially regulated on mRNA level upon hsFLT1 overexpression.

The enlargement of maternal sinusoids in the labyrinthine compartment in the FGR hom and het placentas could be due to a stasis of maternal blood conditioned by a reduced fetal vascular system that increases maternal blood pressure as a possible reactive response to the necessity to fulfill the nutrient requirements of the fetus. Although maternal hypertension and pathological uteroplacental blood flow have not yet been confirmed in our model, this mechanism resembles PE symptoms in humans.

hsFLT1 exerts a strong influence on labyrinth differentiation combined with a decrease of syncytiotrophoblast markers in relation to placental hsFLT1 levels, such as the glucose-diffusion channel *Cx26* and the differentiation-promoting transcription factor *Gcm1*. Gcm1 is one of the leading transcriptional factors during labyrinthine differentiation; it is expressed by a subset of chorionic trophoblast cells and defines the places at which branch points of fetal vessels in the labyrinth will form (41). The reduced transcript expression of markers for the transporting trophoblast fit very well with the FGR phenotype of the fetuses with the various hsFLT1 levels. In addition, the markers for the spongiotrophoblast and glycogen cells, which are mainly responsible for the production of endocrine factors and regulation, have been shown to be upregulated upon hsFLT1 expression; these markers include prolactin *Prl3d1*, as a marker for P-TGCs, and *Cx31*, and *Tpbpa*. This indicates in addition an altered differentiation of the spongiotrophoblast upon hsFLT1 overexpression. The dysregulation of labyrinthine markers in the hsFLT1/rtTA placentas upon hsFLT1 induction in pregnant mice was in accordance with the expression levels of placental nutrient transporters. Transcript levels of glucose transporters *Glut-1* and *Glut-3*, fatty acid transporters *Fabp3* and *Cd36*, and amino acid transporters *Slc38a1* and *Slc38a2* were reduced in hsFLT1–expressing placentas, with the strongest decrease in fatty acid transporters. These alterations strengthened the hypothesis of a negative effect of hsFLT1-expression on placental nutrient transport, leading to a reduction in the transport of nutrients to the fetus. The reduction in the expression of all types of nutrient transporters and of syncytiotrophoblast markers, as well as the reduction in labyrinthine size indicated a reduction in the number of trophoblast cells in the labyrinth but not a downregulation in the expression of these markers per cell. These observations agree with the findings of previous studies using sFLT1 overexpressing mice, which showed a reduction in the size of the labyrinthine compartment (18, 19), and with those of studies using a Plgf knockout mouse model (42) or a Gcm1-deficient mouse model (41, 43).

Thus, we suggest that, in transgenic hsFLT1/rtTA mice, placental function is seriously impaired by hsFLT1 because of a possible deficiency in the placental exchange of nutrients, in particular fatty acid transport. Lipids as central precursors of bioactive molecules are essential for fetal brain development and fetal weight gain (44–46).

Indeed, the metabolic profile of the serum of hsFLT1/rtTA dams showed a change in the metabolome upon hsFLT1 expression, a finding that indicates an accumulation of lysophosphatidylcholines and phosphatidylcholines in the serum of these dams. These findings fitted to the observed reduced level of expression of fatty acid transporters and placental alkaline phosphatases, an enzyme which dephosphorylates phospholipids, which is necessary before transport of the lipids via the placenta (20). Accumulation of phosphatidylcholines in maternal serum and reduction of placental Fabp3 transcript level was also shown in a sFlt-1 overexpressing adenovirus mouse model by Stojanovska et al. (47). Thus, there is an association between reduced placental nutrient transporter expressions with identified protein classes as a result of a dysfunctional placenta. However, we cannot exclude that other altered signaling pathways not involved in placental transport could contribute in elevation of circulating lipids. That FGR is associated with changes in the plasma metabolome, especially amino acid and fatty acid metabolism, has also been shown by other studies, including studies using a maternal diet mouse model (26, 48).

PE with or without FGR is well-known for its long-term consequences for the fetuses (49). Changes in DNA methylation have been proposed to mediate these long-term effects on the fetus (50). Our model of lentiviral sFLT1 overexpression showed methylation of *Igf2*, an imprinted epigenetically regulated gene, and of *H19*, which is co-localized with *Igf2*. Igf2 transcription is dependent on the methylation status of *H19-ICR*, which is located upstream of the H19 promoter (51). In addition, the average methylation of *LINE1* elements was used as a proxy for DNA methylation in general and can therefore serve as an indicator of total DNA methylation levels (52). The use of the transgenic mouse model overexpressing only hsFLT1 did not lead to further changes in the methylation pattern. The current study found only minor changes in DNA methylation, with only one change in *LINE1* and no changes in *H19* or *Igf2*. This finding may be related to the fact that we induced ubiquitous hsFLT1 expression in both the dam and the fetus, and this ubiquitous expression resulted in much higher levels of hsFLT1 at midgestation, unlike the more pathophysiological levels observed in human PE, in which only the placenta overexpresses sFLT1, as in the lentiviral model (19). Moreover, it is tempting to speculate that under the severe conditions observed here we measured methylation in the surviving cell population; thus, there may be an experimental bias by selective survival.

Taken together, the impaired placental development shown in these hsFLT1/rtTA mice ultimately leads to placental insufficiency and FGR. Changes in the transport mechanism of lipids and other nutrients, in combination with a reduction in the placental vascular system, could have caused the strong decrease in fetal weight during pregnancy found in our study. Thus, we speculate that the alterations triggered by the increase in anti-angiogenesis brought about by hsFLT1 expression not only may strongly affect the maternal cardiovascular system, as shown by Mosca et al. (53, 54), but also may adversely affect the fetus by altering development and function of the placenta as the first fetal organ, increasing the risk of cardiovascular and neurological diseases later in life (55).

## Conclusion

We introduced a novel stable and reproducible transgenic hsFLT1/rtTA FGR mouse model, in which hsFLT1 overexpression can be ubiquitously induced at several time points during murine pregnancy. Using this model, we were able to discriminate between the effects of hsFLT1 overexpression by the dam and the placenta/fetus and those of overexpression by the dam alone. FGR has developed in all hsFLT1 expressing groups at term, and its severity depended on the hsFLT1 expression strength. In the present study we focused primarily on the consequences of hsFLT1 overexpression on placental development, with a special focus on placental vascularization and nutrient transport. The results indicate the importance of the Vegf/sFlt-1 system in placental development in stages following 7.5 dpc in mice. hsFLT1 inhibits placental differentiation, especially inhibiting fetal capillary branching by reducing Vegf signaling and inducing apoptosis in fetal ECs. Over time, this inhibition could lead to a stasis of maternal blood, promoting dilatation of the maternal sinusoids because of impairment of the fetal vessel system. The altered placental morphology ultimately results in uteroplacental insufficiency, including reduced nutrient transport (predominantly affecting the fatty acid supply), which leads to FGR.

Currently we are determining whether these hsFLT1/rtTA mice also exhibit typical symptoms of PE. This improved model can serve as a tool for further molecular biological investigations of sFLT1–mediated pathophysiology in PE with FGR as well as for the development of maternal diseases. Furthermore, it could yield new treatment options for PE and could be used in follow-up studies of fetal and maternal outcome.

## Ethics Statement

All animal experiments were approved and performed in accordance with the guidelines of the University Hospital Essen, Germany, and with local government approval by the State Agency for Nature, Environment and Consumer Protection, North Rhine-Westphalia (LANUV).

## Author Contributions

AG, EK, and EW initiated and organized the study. ND and HS designed the hsFLT1 mouse strain. AG, EK, RV, and EW designed the experiments. RV performed the experiments, analyzed the data, and prepared the figures and tables. TP and RV-S performed epigenetic analysis, including discussion of the epigenetic data. AG, FH, AK, RK, TP, VS, RV, and EW discussed the data. AG, RV, and EW wrote the manuscript. All authors contributed to manuscript revision and read and approved the submitted version.

### Conflict of Interest Statement

The authors declare that the research was conducted in the absence of any commercial or financial relationships that could be construed as a potential conflict of interest.

## Supplementary Material

Figure S1 Comparison of fetal **(A)** and placental weight **(B)** as well as placental efficiency **(C)** of hsFLT1 expression starting at gestational day (dpc) 7.5 or 10.5 until 18.5 dpc in the hsFLT1/rtTA mouse-model. **(A)** fetal weights: Controls 7.5, *n* = 13, 10.5, *n* = 25; Dox controls 7.5, *n* = 32, 10.5, *n* = 25; FGR wt 7.5, *n* = 12, 10.5, *n* = 4; FGR het 7.5, *n* = 3, 10.5, *n* = 19; FGR hom 7.5, *n* = 3, 10.5, *n* = 12. **(B)** Placental weights: Controls 7.5, *n* = 11, 10.5, *n* = 25; Dox controls 7.5, *n* = 30, 10.5, *n* = 20; FGR wt 7.5, *n* = 12, 10.5, *n* = 3; FGR het 7.5, *n* = 3, 10.5, *n* = 18; FGR hom 7.5, *n* = 4, 10.5, *n* = 20. **(C)** Placental efficiency: Controls 7.5, *n* = 11, 10.5, *n* = 23; Dox controls 7.5, *n* = 30, 10.5, *n* = 20; FGR wt 7.5, *n* = 12, 10.5, *n* = 3; FGR het 7.5, *n* = 3, 10.5, *n* = 17; FGR hom 7.5, *n* = 3, 10.5, *n* = 17. Data is presented as median and interquartile range. Two-Way ANOVA with Bonferroni post tests revealed no changes between the starting time points 7.5 and 10.5 dpc in each experimental condition (*p* > 0.05).

Figure S2 Sex-specific differences in fetal weight and placental efficiency in the hsFLT1/rtTA mouse model. **(A)** Body weight of mouse fetuses at day 18.5 post coitum (dpc) was lower in females expressing human soluble fms-like tyrosine kinase-1 (hsFLT1) than in males expressing hsFLT1. **(B)** In addition, reduced placental efficiency affected female fetuses more frequently than male fetuses. ^*^*p* < 0.05; ^***^*p* < 0.001 as determined by the Kruskal–Wallis test with Dunn's *post hoc* test. *P*-values of each fetal growth restriction (FGR) group are shown only in contrast to control group fetuses of the same sex. **(C)** Percentages show the distributions of males and females within the experimental groups. The FGR group contained the fewest males (25.8%) in comparison to the number of females (48.5%) but also contained the highest number of fetuses of unknown gender (25.7%), a finding that is mostly due to technical reasons because of the high degree of degraded DNA in the retarded cyanotic fetuses. In comparison, the control group contained 40.5% males, 52.4% females, and 7.1% fetuses of unknown gender; the doxycycline (Dox) control group contained 65.5% males, 32.8% females, and only 1.7% fetuses of unknown gender.

Figure S3 Correlations of fetal weight and placental efficiency with either placental hsFLT1 transcript level **(A)** or fetal hsFLT1 transcript level **(B,C)** in the hsFLT1/rtTA mouse model. **(A)** Reduction in fetal body weight was negatively correlated with fetal expression of human soluble fms-like tyrosine kinase-1 (hsFLT1) (*r* = −0.6691; *p* = 0.0033). Placental efficiency exhibited a moderate negative correlation with the expression of fetal hsFLT1 (*r* = −0.4059; *p* = 0.1188) **(B)** and a weak negative correlation with the expression of placental hsFLT1 (*r* = −0.2439; *p* = 0.24) **(C)**.

Figure S4 Analysis of the expression of important placental marker genes in the hsFLT1/rtTA mouse model. **(A)** Heat-map representation of the results of quantitative reverse transcription polymerase chain reaction (qRT-PCR) of 34 marker genes detected differences in placental gene expression between fetal growth restriction (FGR) homozygous (*n* = 7) or FGR heterozygous (*n* = 15) placentas and control (*n* = 12) or doxycycline (Dox) control (*n* = 13) placentas. The expression of human soluble fms-like tyrosine kinase-1 (hsFLT1) changed the expression of 22 of 34 genes, as shown by one-way analysis of variance (ANOVA; color-coding intensity in the red spectrum shows an increase in the expression of a marker gene, and color intensity in the blue spectrum shows a decrease in the expression of a given marker gene). **(B)** Protein levels of differentially regulated angiogenesis-related factors (Tissue Factor, Serpin E1, and Serpin F1) detected with Proteome Profiler Angiogenesis Antibody Array. All three factors were upregulated upon hsFLT1 expression in FGR hom (*n* = 5) and het (*n* = 5) placentas compared to control (*n* = 5) and Dox control (*n* = 5) placentas. Data is presented as mean ± standard deviation.

Figure S5 Characterization of average DNA methylation of insulin-like growth factor two (*Igf2*) differentially methylated region two (*DMR2*) **(A)**, H19 imprinting control region (*H19-ICR*) **(B)** and global DNA methylation by long interspersed element one (*LINE1*) **(C)** in placentas of the human soluble fms-like tyrosine kinase reverse tetracycline-controlled transactivator (hsFLT1/rtTA) mouse model. Samples were obtained from complete placentas. Methylation levels were analyzed by pyrosequencing. The following experimental groups were analyzed: fetal growth restriction homozygous (FGR hom; *n* = 4); FGR heterozygous (het; *n* = 7); FGR wild-type (wt; *n* = 7) in various shades of red; and control (*n* = 10) and doxycycline (Dox) control (*n* = 6) groups in different shades of gray. Data are presented as means ± standard error of the mean. ^*^*p* < 0.05, as determined by the Kruskal–Wallis test with Dunn's *post hoc* test.
