# Supplementary figures and images for: Human sFLT1 Leads to Severe Changes in Placental Differentiation and Vascularization in a Transgenic hsFLT1/rtTA FGR Mouse Model
Source: Front Endocrinol (Lausanne). 2019 Mar 21;10:165. doi: 10.3389/fendo.2019.00165 (PMC6437783; doi:10.3389/fendo.2019.00165)

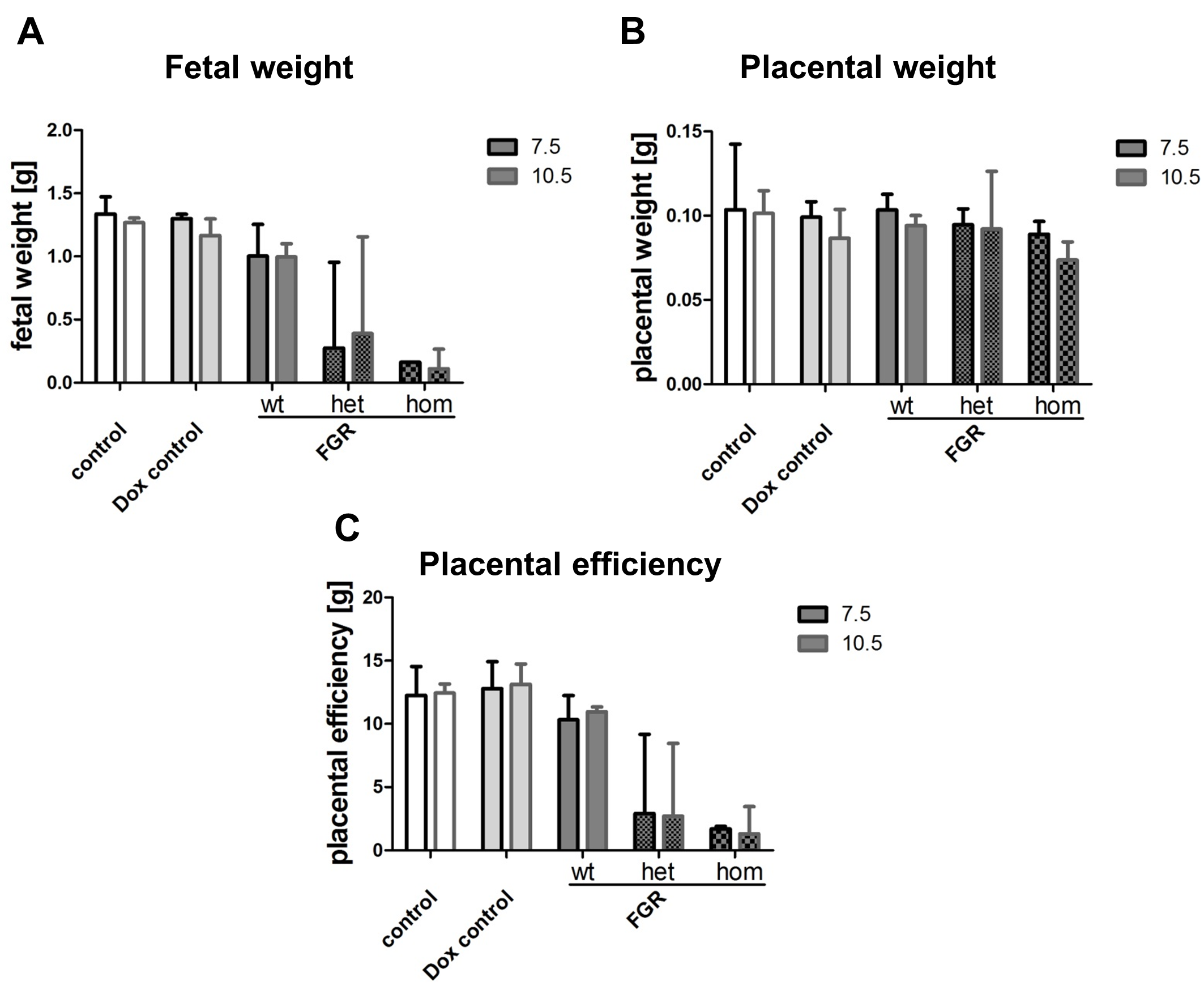

Supplement: Figure S1 — Comparison of fetal (A) and placental weight (B) as well as placental efficiency (C) of hsFLT1 expression starting at gestational day (dpc) 7.5 or 10.5 until 18.5 dpc in the hsFLT1/rtTA mouse-model. (A) fetal weights: Controls 7.5, n = 13, 10.5, n = 25; Dox controls 7.5, n = 32, 10.5, n = 25; FGR wt 7.5, n = 12, 10.5, n = 4; FGR het 7.5, n = 3, 10.5, n = 19; FGR hom 7.5, n = 3, 10.5, n = 12. (B) Placental weights: Controls 7.5, n = 11, 10.5, n = 25; Dox controls 7.5, n = 30, 10.5, n = 20; FGR wt 7.5, n = 12, 10.5, n = 3; FGR het 7.5, n = 3, 10.5, n = 18; FGR hom 7.5, n = 4, 10.5, n = 20. (C) Placental efficiency: Controls 7.5, n = 11, 10.5, n = 23; Dox controls 7.5, n = 30, 10.5, n = 20; FGR wt 7.5, n = 12, 10.5, n = 3; FGR het 7.5, n = 3, 10.5, n = 17; FGR hom 7.5, n = 3, 10.5, n = 17. Data is presented as median and interquartile range. Two-Way ANOVA with Bonferroni post tests revealed no changes between the starting time points 7.5 and 10.5 dpc in each experimental condition (p > 0.05). [file Image_1.TIF]

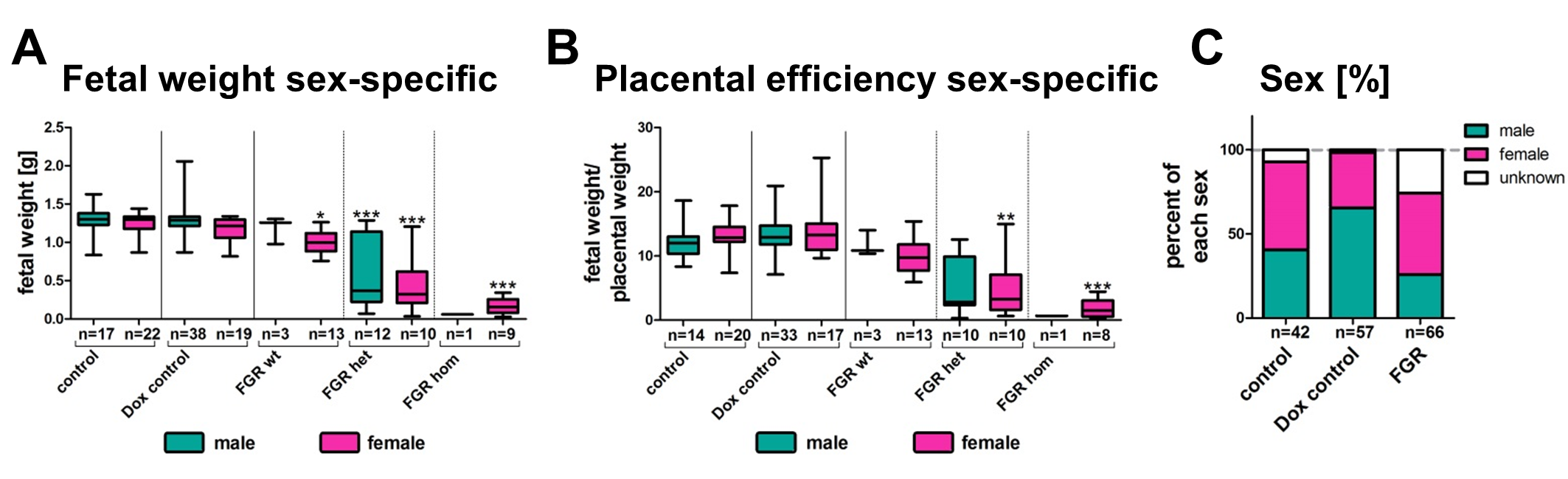

Supplement: Figure S2 — Sex-specific differences in fetal weight and placental efficiency in the hsFLT1/rtTA mouse model. (A) Body weight of mouse fetuses at day 18.5 post coitum (dpc) was lower in females expressing human soluble fms-like tyrosine kinase-1 (hsFLT1) than in males expressing hsFLT1. (B) In addition, reduced placental efficiency affected female fetuses more frequently than male fetuses. *p < 0.05; ***p < 0.001 as determined by the Kruskal–Wallis test with Dunn's post hoc test. P-values of each fetal growth restriction (FGR) group are shown only in contrast to control group fetuses of the same sex. (C) Percentages show the distributions of males and females within the experimental groups. The FGR group contained the fewest males (25.8%) in comparison to the number of females (48.5%) but also contained the highest number of fetuses of unknown gender (25.7%), a finding that is mostly due to technical reasons because of the high degree of degraded DNA in the retarded cyanotic fetuses. In comparison, the control group contained 40.5% males, 52.4% females, and 7.1% fetuses of unknown gender; the doxycycline (Dox) control group contained 65.5% males, 32.8% females, and only 1.7% fetuses of unknown gender. [file Image_2.TIF]

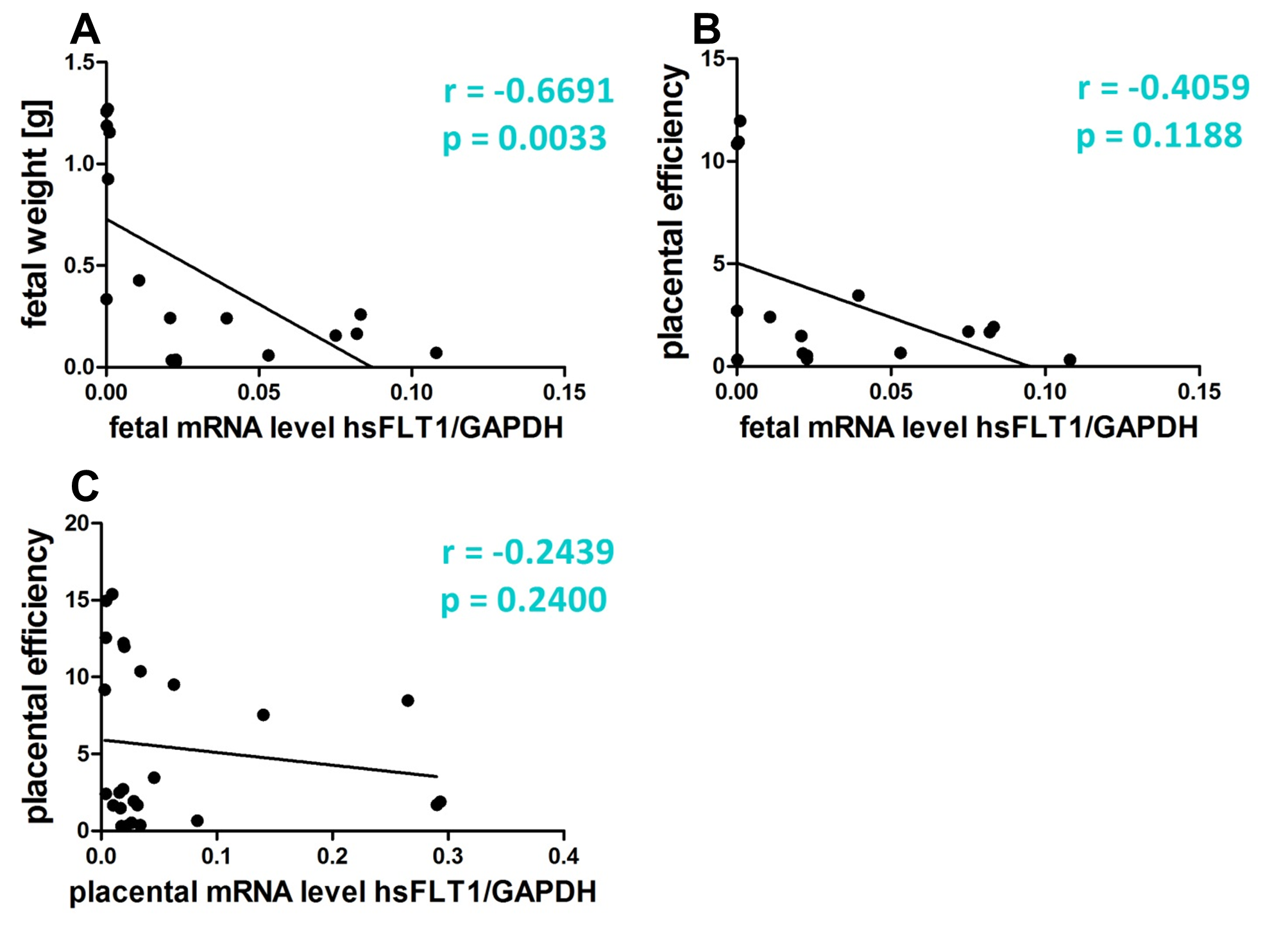

Supplement: Figure S3 — Correlations of fetal weight and placental efficiency with either placental hsFLT1 transcript level (A) or fetal hsFLT1 transcript level (B,C) in the hsFLT1/rtTA mouse model. (A) Reduction in fetal body weight was negatively correlated with fetal expression of human soluble fms-like tyrosine kinase-1 (hsFLT1) (r = −0.6691; p = 0.0033). Placental efficiency exhibited a moderate negative correlation with the expression of fetal hsFLT1 (r = −0.4059; p = 0.1188) (B) and a weak negative correlation with the expression of placental hsFLT1 (r = −0.2439; p = 0.24) (C). [file Image_3.TIF]

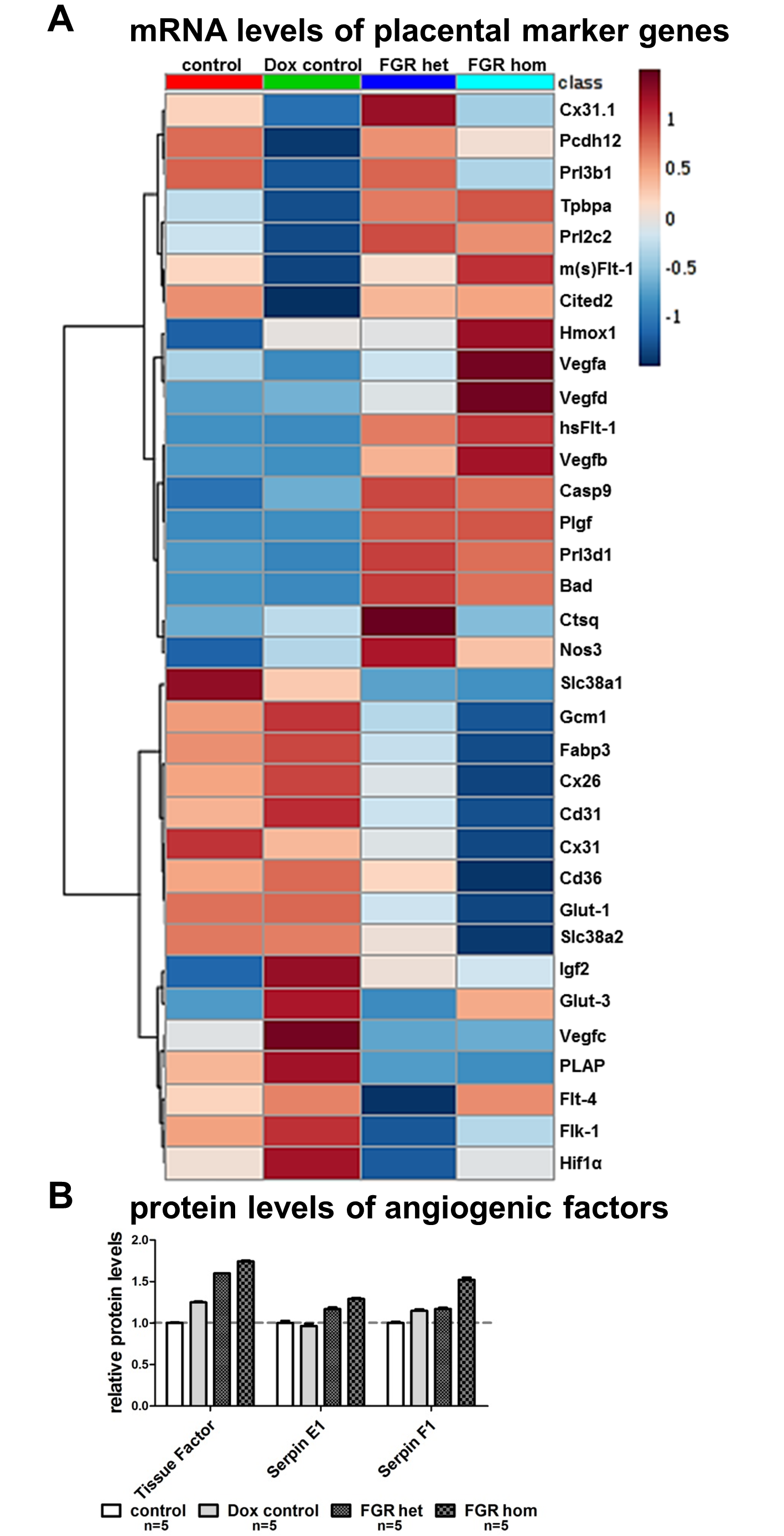

Supplement: Figure S4 — Analysis of the expression of important placental marker genes in the hsFLT1/rtTA mouse model. (A) Heat-map representation of the results of quantitative reverse transcription polymerase chain reaction (qRT-PCR) of 34 marker genes detected differences in placental gene expression between fetal growth restriction (FGR) homozygous (n = 7) or FGR heterozygous (n = 15) placentas and control (n = 12) or doxycycline (Dox) control (n = 13) placentas. The expression of human soluble fms-like tyrosine kinase-1 (hsFLT1) changed the expression of 22 of 34 genes, as shown by one-way analysis of variance (ANOVA; color-coding intensity in the red spectrum shows an increase in the expression of a marker gene, and color intensity in the blue spectrum shows a decrease in the expression of a given marker gene). (B) Protein levels of differentially regulated angiogenesis-related factors (Tissue Factor, Serpin E1, and Serpin F1) detected with Proteome Profiler Angiogenesis Antibody Array. All three factors were upregulated upon hsFLT1 expression in FGR hom (n = 5) and het (n = 5) placentas compared to control (n = 5) and Dox control (n = 5) placentas. Data is presented as mean ± standard deviation. [file Image_4.TIF]

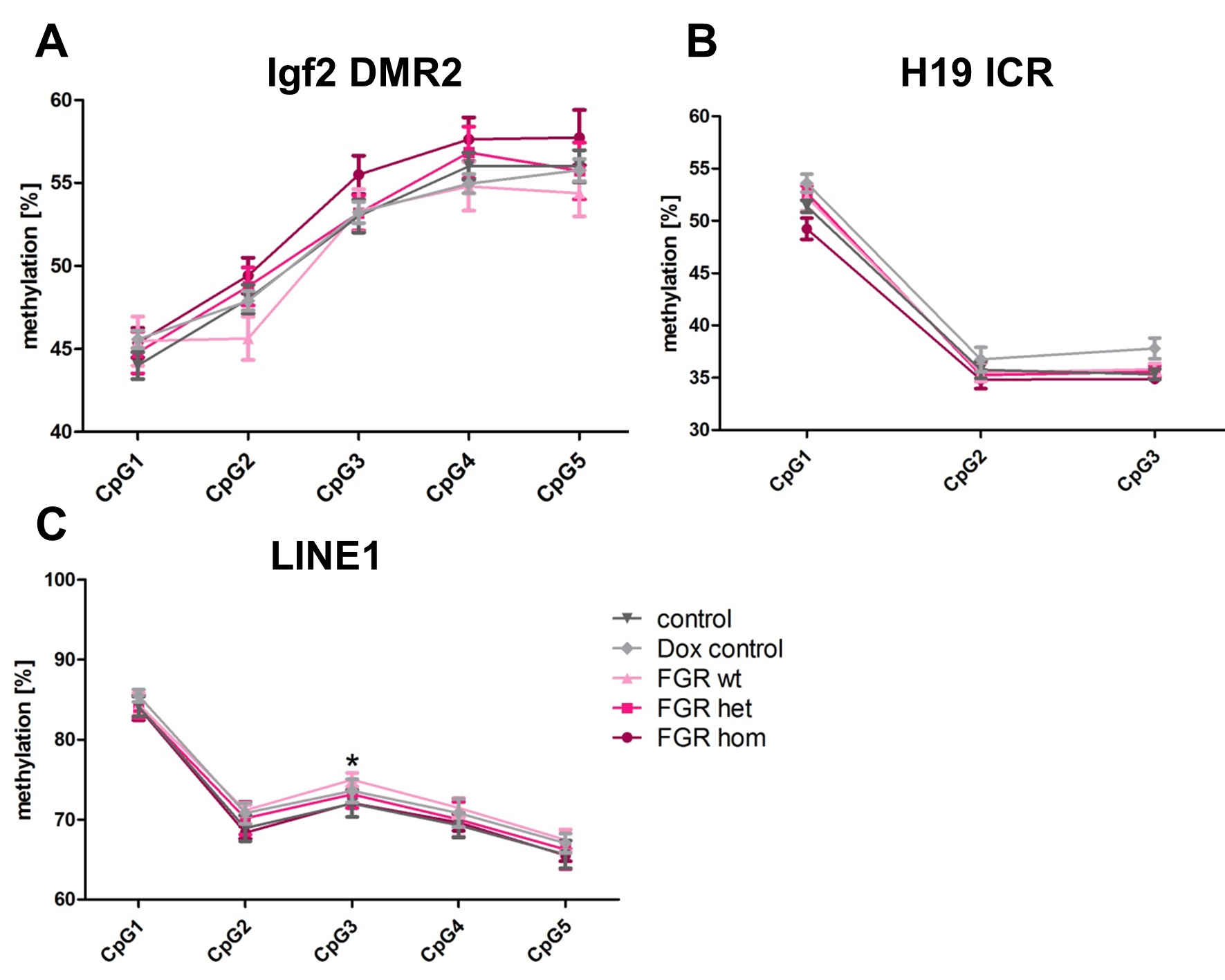

Supplement: Figure S5 — Characterization of average DNA methylation of insulin-like growth factor two (Igf2) differentially methylated region two (DMR2) (A), H19 imprinting control region (H19-ICR) (B) and global DNA methylation by long interspersed element one (LINE1) (C) in placentas of the human soluble fms-like tyrosine kinase reverse tetracycline-controlled transactivator (hsFLT1/rtTA) mouse model. Samples were obtained from complete placentas. Methylation levels were analyzed by pyrosequencing. The following experimental groups were analyzed: fetal growth restriction homozygous (FGR hom; n = 4); FGR heterozygous (het; n = 7); FGR wild-type (wt; n = 7) in various shades of red; and control (n = 10) and doxycycline (Dox) control (n = 6) groups in different shades of gray. Data are presented as means ± standard error of the mean. *p < 0.05, as determined by the Kruskal–Wallis test with Dunn's post hoc test. [file Image_5.TIF]
